# Association of DNA Methylation Patterns in 7 Novel Genes With Ischemic Stroke in the Northern Chinese Population

**DOI:** 10.3389/fgene.2022.844141

**Published:** 2022-04-11

**Authors:** Hongwei Sun, Jia Xu, Bifeng Hu, Yue Liu, Yun Zhai, Yanyan Sun, Hongwei Sun, Fang Li, Jiamin Wang, Anqi Feng, Ying Tang, Jingbo Zhao

**Affiliations:** ^1^ Department of Neurology, The First Affiliated Hospital of Harbin Medical University, Harbin, China; ^2^ Department of Epidemiology, School of Public Health, Harbin Medical University, Harbin, China

**Keywords:** ischemic stroke, epigenome-wide association study, DNA methylation, gene ontology, cadherins

## Abstract

**Background:** Ischemic stroke is a highly complex disorder. This study aims to identify novel methylation changes in ischemic stroke.

**Methods:** We carried out an epigenome-wide study of ischemic stroke using an Infinium HumanMethylation 850K array (cases:controls = 4:4). 10 CpG sites in 8 candidate genes from gene ontology analytics top-ranked pathway were selected to validate 850K BeadChip results (cases:controls = 20:20). We further qualified the methylation level of promoter regions in 8 candidate genes (cases:controls = 188:188). Besides, we performed subgroup analysis, dose-response relationship and diagnostic prediction polygenic model of candidate genes.

**Results:** In the discovery stage, we found 462 functional DNA methylation positions to be associated with ischemic stroke. Gene ontology analysis highlighted the “calcium-dependent cell-cell adhesion via plasma membrane cell adhesion molecules” item, including 8 candidate genes (*CDH2/PCDHB10/PCDHB11/PCDHB14/PCDHB16/PCDHB3/PCDHB6/PCDHB9*). In the replication stage, we identified 5 differentially methylated loci in 20 paired samples and 7 differentially methylated genes (*CDH2/PCDHB10/PCDHB11/PCDHB14/PCDHB16/PCDHB3/PCDHB9*) in 188 paired samples. Subgroup analysis showed that the methylation level of above 7 genes remained significantly different in the male subgroup, large-artery atherosclerosis subgroup and right hemisphere subgroup. The methylation level of each gene was grouped into quartiles, and Q4 groups of the 7 genes were associated with higher risk of ischemic stroke than Q1 groups (*p* < 0.05). Besides, the polygenic model showed high diagnostic specificity (0.8723), sensitivity (0.883), and accuracy (0.8777).

**Conclusion:** Our results demonstrate that DNA methylation plays a crucial part in ischemic stroke. The methylation of these 7 genes may be potential diagnostic biomarker for ischemic stroke.

## Introduction

Stroke is a devastating disease due to its high morbidity, disability, and recurrence. The prevalence of stroke is increasing over time, and the affected population is becoming younger (Katan and Luft, 2018). Ischemic stroke (IS) is the dominant subtype of stroke with a proportion more than 80% (Ajoolabady et al., 2021). Many of the environmental and genetic risk factors are associated with IS, and the genetic risk is believed to be in the order of 37.9%. However, genetic variants associated with IS found to date only account for 5–10% of that genetic risk (Bevan et al., 2012; Krupinski et al., 2017), which suggests that more associated heritable risk factors have not yet been discovered. Epigenetic modifications is one of these possible heritable changes (Mahjoubin-Tehran et al., 2021).

Epigenetics refer to chemical modifications of DNA structure without affecting the DNA sequence that may provide a link between environment and gene expression (Majnik and Lane, 2014). Generally, epigenetic modifications of gene expression occur by three main forms: DNA methylation, histone modification and microRNA expression (Schiano et al., 2020; Liu et al., 2020). As a major type of epigenetic process, DNA methylation mainly occurs at the cytosine of a cytosine-phosphate-guanine (CpG) dinucleotide forming 5-methylcytosine, which can inhibit gene expression through transcriptional silencing (Bird, 2007). DNA methylation is a crucial epigenetic mechanism involved in normal and pathological cellular processes (Krupinski et al., 2017).

The current epigenome-wide studies revealed that DNA methylation plays a vital part in the pathogenesis and recurrence of IS (Soriano-Tárraga et al., 2020; Davis Armstrong et al., 2018; Gómez-Úriz et al., 2015; Shen et al., 2019), which have mainly been conducted in American and European populations (Soriano-Tárraga et al., 2020; Davis Armstrong et al., 2018; Gómez-Úriz et al., 2015). An epigenetic study in a Chinese population revealed that hypomethylation of the *MTRNR2L8* gene is associated with IS (Shen et al., 2019). It is undeniable that this study is significant; however, it focused on the large-artery atherosclerosis stroke, a subgroup of IS, which cannot fully demonstrate the role of DNA methylation in IS. Thus far, epigenome-wide alterations of IS have not been systematically investigated in a Chinese population.

In our research, we designed a two-stage case-control study to perform integrated analysis of genome-wide DNA methylation profiles to identify novel candidate genes and pathways for IS in a Chinese population.

## Materials and Methods

### Ethical Approval

We performed this study according to the ethical standards laid down in the 1964 Declaration of Helsinki and its later amendments. The study procedure was approved by the Ethics Committee of the First Affiliated Hospital of Harbin Medical University. All participants voluntarily gave written informed consent.

### Study Population

**FIGURE 1:**
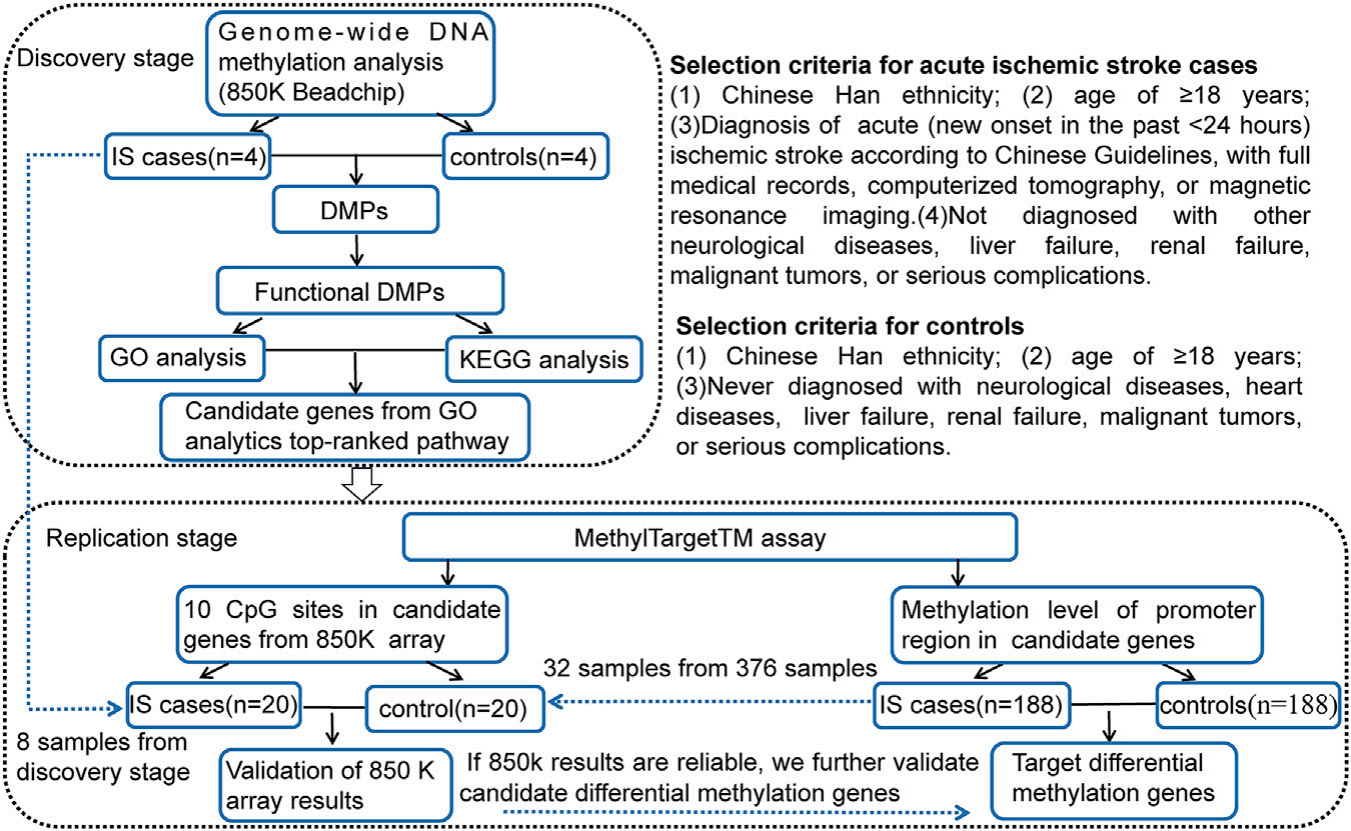
Two-stage case-control study design and selection criteria for cases and controls. IS, ischemic stroke; DMPs, DNA methylation positions; GO, gene ontology; KEGG, Kyoto Encyclopedia of Genes and Genomes; CpG, cytosine phosphate guanine.

In this study, a two-stage case-control DNA methylation study was designed, including discovery and replication analyses. The sample consisted of 192 IS patients and 192 age-(±3 years) and sex-matched controls from the First Affiliated Hospital of Harbin Medical University. Figure 1 demonstrates the study design and selection criteria for cases and controls. The control group was from healthy people who underwent physical examination or patients hospitalized from the First Affiliated Hospital of Harbin Medical University at the same period.

### Data and Sample Collection

Data for subjects included questionnaire, laboratory investigations, and clinical characteristics. We collected data using a questionnaire related to demographic information (such as gender, age, and marital status), lifestyle habits (such as tobacco smoking and alcohol drinking), and past medical history (hypertension and type 2 diabetes mellitus) of the participants (Qin et al., 2019).

Laboratory investigations were done at the First Affiliated Hospital of Harbin Medical University. We collected blood samples in tubes with clot activator and gel separator from all participants in the morning after fasting and measured using an auto biochemical analyzer (Biobase BK-600; Shandong, China). The levels of total triglyceride (TG), total cholesterol (TC), high-density lipoprotein (HDL), and low-density lipoprotein (LDL) was collected from all participants.

Clinical characteristics included Trial of Org 10172 in Acute Stroke Treatment (TOAST) type and stroke location. Stratification analysis by TOAST criteria was performed for large-artery atherosclerosis (LAA), cardioembolism (CE), small vessel disease (SVD), other determined cause (ODC), and undetermined (UND) aetiologies. Stroke location was categorized as anterior, posterior, and both anterior and posterior circulation. Anterior circulation was further subdivided as left and right hemisphere based on computerized tomography or magnetic resonance imaging scans.

Two milliliters of blood samples were collected in EDTA tubes from all participants and stored at −80°C until DNA extraction.

### Discovery Stage: Genome-Wide DNA Methylation Profiles

Genomic DNA was extracted from the leukocytes in the peripheral blood using an DNA Methylation kit (Zymo, Irvine, CA), following the protocol (Genesky Biotechnologies Inc., Shanghai, China). Genome-wide DNA methylation was assessed using Infinium Human Methylation 850K BeadChip (Illumina Inc., San Diego, CA, United States)). More than 853,000 CpG sites were included in each chip. CpG sites containing documented single-nucleotide polymorphisms and mapping to X and Y chromosomes were removed to avoid potential confounding by single-nucleotide polymorphisms and gender.

### Discovery Stage: Bioinformatics Analysis of DNA Methylation Profiles

To screen functional DNA methylation positions (DMPs), DMPs located in promoter regions (upstream or 5′untranslated region, 5′UTR), with |methylation difference|>0.1 and *p* < 0.05 were selected. We performed Gene ontology (GO, http://www.geneontology.org) and the Kyoto Encyclopedia of Genes and Genomes (KEGG, http://www.kegg.jp/) pathway enrichment analysis to clarify the function and biological pathways of differential methylation loci. Through GO enrichment analysis, the differentially methylated genes were classified according to cellular component, molecular function and biological process. In GO and KEGG enrichment analysis, *p* < 0.05 was significantly enriched by differential methylation loci-related genes.

### Replication Stage: MethylTargetTM Assay

**FIGURE 2:**
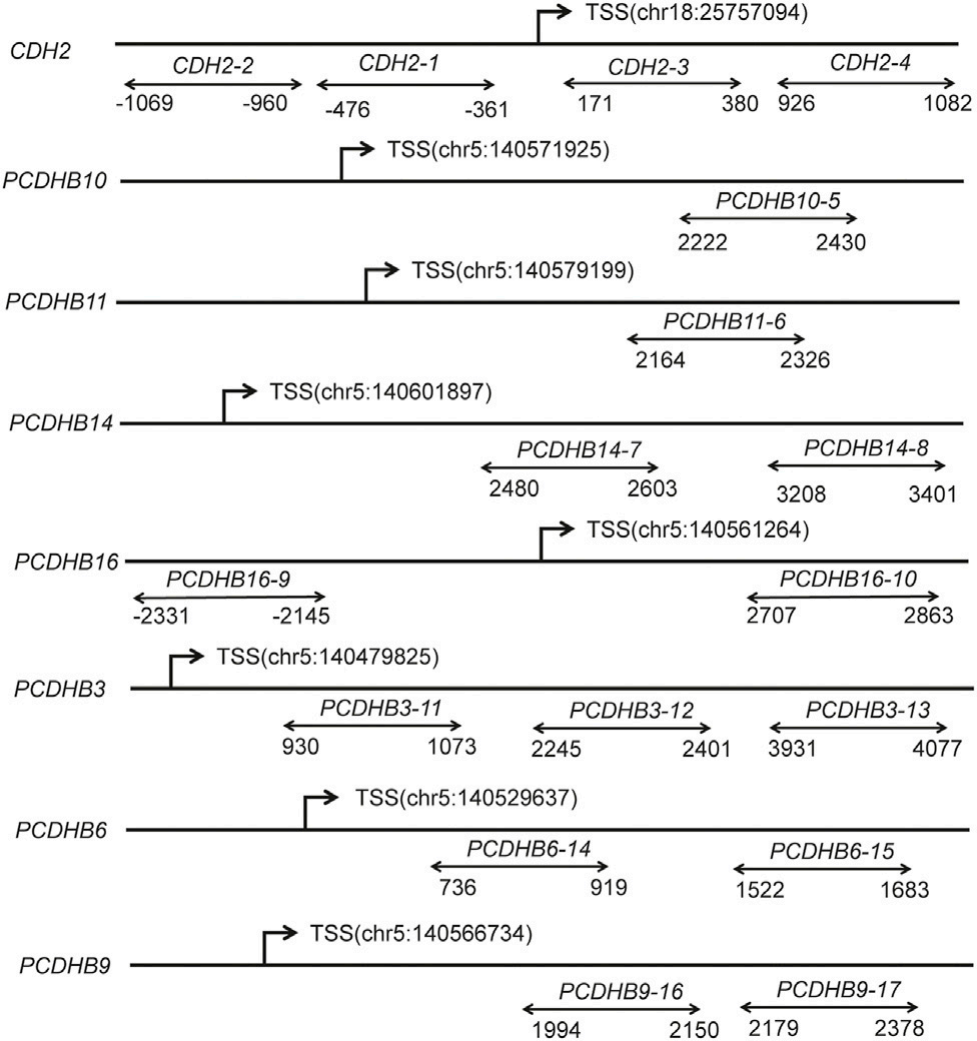
Diagram of the structure of the 8 genes and 17 CpG islands. The line represents the gene, and double arrows represent CpG islands. CpG, cytosine phosphate guanine; TSS, transcription start sites; Chr, chromosome.

Considering the *p*-values and biological functions of the genes, we selected 8 candidate genes from GO analytics top-ranked pathway including 10 CpG sites to validate 850 K BeadChips results using MethylTargetTM assay, which was a multi-targeted CpG methylation analysis method based on next-generation sequencing (Wan et al., 2021). As the 10 CpG sites in 850 K BeadChips results are not top-ranked loci and FDR *p* values of the 10 CpG sites are not statistically significant, we replicated 10 CpGs in a small replication sample (20 cases:20 matched controls, 8 samples from DNA methylation chip analysis and 32 samples from validation analysis). Moreover, we further qualified the methylation level of promoter regions in 8 candidate genes, containing 17 CpG islands and 308 CpG sites, using a large replication sample (188 cases:188 individually matched controls). The locations of the 17 CpG islands in 8 genes are shown in Figure 2. The primers were carefully designed (Additional File S1).

### Validation of the mRNA Expression of Differentially Methylated Genes

The dataset GSE22255 was acquired from the GEO database (https://www.ncbi.nlm.nih.gov/geo/) and was utilized as the validation sample. Gene expression profiling was performed in peripheral blood of 20 IS patients and 20 sex- and age-matched controls. From this dataset, the downloaded data format was MINIML. Box plots of expression were drawn by the R software package ggplot2, and principal component analysis graphs were drawn by the R software package ggord. The Wilcoxon rank-sum test was used to detect whether the mRNA expression of differentially methylated genes was different between stroke patients and controls.

### Statistical Analysis

Quantitative variables were reported as the mean and standard deviation, while qualitative variables were expressed as frequencies and percentages (%). The normality of quantitative variables was assessed using the Shapiro–Wilk test.

First, we compared the differences of baseline demographic characteristics, lifestyle habits, medical history, and lipid levels between IS cases and controls. The differences of the categorical variables were measured with a chi-square test. We performed a paired *t* test for the normal distributed continuous variables. Non-parametric test was used for the skewed distributed continuous variables.

Second, the methylation status was compared between IS cases and marched controls using conditional logistic regression analysis. Smoking, drinking, medical history of diabetes mellitus and hypertension, and lipid levels were added as adjusted factors. The association of the methylation level of genes was assessed using Pearson’s correlation. All subjects were classified into four groups (Q1, Q2, Q3, and Q4) based on the quartile value of methylation level. Q1 group having the lowest methylation level is regarded as the reference group, and all other groups (Q2 having 25–50% of the values, Q3 having 50–75%, and Q4 having 75–100%) were compared with Q1 group (Qin et al., 2019).

The methylation level of each CpG site was measured as the percentage of the methylated cytosines over total tested cytosines. The average methylation level of all CpG sites was worked out as the methylation level of the DNA segments and genes. Results were considered statistically significant when the *p* values were less than 0.05. All statistical analyses were performed with the SAS version 9.4 for Windows (SAS Institute, Inc., Cary, NC).

## Results

### Characteristics of Participants

The distribution of characteristics for the participants are presented in Table 1. Compared with controls, IS patients in validation stage had a higher prevalence of smoking, drinking and hypertension (*p* < 0.0001), and higher level of triglyceride (*p* = 0.0288).

**TABLE 1:** Characteristics of participants.

	DNA methylation chip analysis		*P*	DNA methylation validation analysis		*P*
	IS (*n* = 4)	Control (*n* = 4)		IS (*n* = 188)	Control (*n* = 188)	
Age	65.2397 ± 2.6561	65.9904 ± 2.9258	0.7171	62.2155 ± 10.6660	62.1466 ± 10.5739	0.9499
Female	2 (50%)	2 (50%)	1.000	44 (23.40%)	44 (23.40%)	1.000
Smoking (%)	1 (25%)	1 (25%)	1.000	106 (56.38%)	41 (21.81%)	<0.0001*
Drinking (%)	1 (25%)	1 (25%)	1.000	91 (48.40%)	39 (20.74%)	<0.0001*
Hypertension (%)	0 (0%)	0 (0%)	1.000	112 (59.57%)	69 (36.70%)	<0.0001*
Diabetes (%)	0 (0%)	0 (0%)	1.000	35 (18.62)	26 (13.83%)	0.2080
Total cholesterol (mmol/L)	5.4900 ± 0.8676	5.1325 ± 0.2749	0.4620	5.4598 ± 4.5605	4.8362 ± 1.0503	0.0691
Triglyceride (mmol/L)	1.7350 ± 0.8916	1.4225 ± 1.0174	0.6604	1.9978 ± 1.3728	1.7139 ± 1.1217	0.0288*
High-density lipoprotein (mmol/L)	1.1650 ± 0.2883	1.5425 ± 0.5916	0.2950	1.2963 ± 0.9580	1.2990 ± 0.4034	0.9715
Low-density lipoprotein (mmol/L)	3.5500 ± 0.4497	2.9450 ± 0.3692	0.0828	3.2304 ± 0.7974	2.9960 ± 1.8316	0.1089

*Statistically significant difference (p < 0.05). IS, ischemic stroke.

### Discovery Stage: Epigenome-Wide Association Study of IS

In the discovery analysis, 2656 DMPs exhibited differences (*p* < 0.05, |methylation difference|>0.1) in DNA methylation between the two groups. The results are shown in another unpublished article (Xu J, et al.). After screening, a total of 462 functional DMPs (located in the promoter region, |methylation difference| > 0.1 and *p* < 0.05) corresponding to 373 genes were selected (Additional File S2). Among them, the majority of the DMPs were in CpG island (26.4%) and N-shore (28.6%). In addition, 87.7% (405 of 462) CpG cites corresponding to 327 genes were found to be hypomethylated in IS cases compared to controls.

### Discovery Stage: Bioinformatics Analysis of DNA Methylation Profiles

**FIGURE 3:**
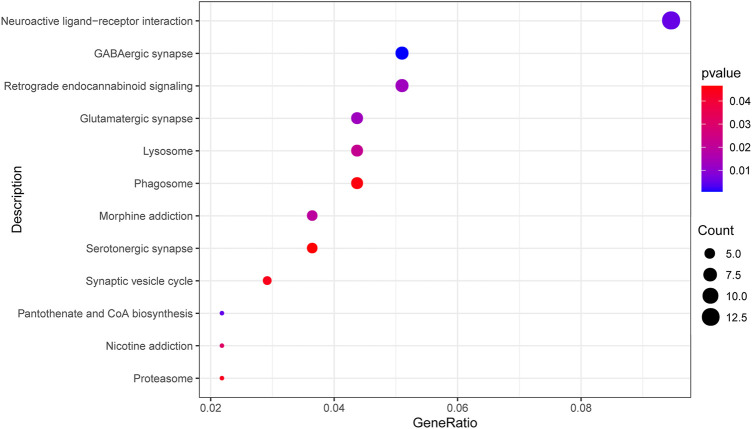
Top 12 enriched KEGG pathways of differential methylation loci-related genes. The dot size represents the number of genes in the pathway, and the dot color represents the enrichment *p* value. KEGG, Kyoto Encyclopedia of Genes and Genomes; GABA, gamma-aminobutyric acid.

We screened and finally included 12 KEGG pathways (*p* < 0.05, Figure 3). A total of 315 significant GO terms (*p* < 0.05) were selected. The top 10 GO items were shown in Table 2. Among them, the “calcium-dependent cell-cell adhesion via plasma membrane cell adhesion molecules” item showed the most significant fold enrichment. There are 8 genes in this item including *CDH2/PCDHB10/PCDHB11/PCDHB14/PCDHB16/PCDHB3/PCDHB6/PCDHB9*, and the 8 genes were selected for replication.

**TABLE 2:** Top 10 enriched GO pathways of differential methylation loci-related genes.

ID	Description	Genes	*P*
BP			
GO:0016339	calcium-dependent cell-cell adhesion via plasma membrane cell adhesion molecules	*PCDHB14/PCDHB11/PCDHB3/PCDHB16/PCDHB9/CDH2/PCDHB10/PCDHB6*	*p* < 0.0001
GO:0007156	homophilic cell adhesion via plasma membrane adhesion molecules	*DSC3/PCDHGA4/PCDHA6/PCDHB14/PCDHGA11/PCDHB11/PCDHB3/PCDHGA1/PCDHA1/PCDHB16/PCDHB9/CDH2/PCDHB7/PCDHB10/PCDHB6*	*p* < 0.0001
GO:0098742	cell-cell adhesion via plasma-membrane adhesion molecules	*DSC3/PCDHGA4/PCDHA6/CBLN1/PCDHB14/PCDHGA11/PCDHB11/PCDHB3/PCDHGA1/PCDHA1/PCDHB16/PCDHB9/CDH2/PCDHB7/PCDHB10/CD164/PCDHB6*	*p* < 0.0001
GO:0007416	synapse assembly	*CBLN1/NRG1/PCDHB14/THBS2/PCDHB11/PCDHB3/PCDHB16/PCDHB9/CDH2/NRXN2/PCDHB7/PCDHB10/PCDHB6*	*p* < 0.0001
GO:0050808	synapse organization	*ANK3/CBLN1/NRG1/PCDHB14/THBS2/CTNND2/CACNB2/PCDHB11/PCDHB3/PCDHB16/PCDHB9/CDH2/NRXN2/PCDHB7/PCDHB10/PCDHB6*	*p* < 0.0001
GO:0048863	stem cell differentiation	*PSMA8/NRG1/KIT/EDN3/TP73/MYOCD/TAL1/PSMD1/PSMB2/SFRP1/SEMA3C/MSX1/YTHDF2*	*p* = 0.0008
CC			
GO:0045211	postsynaptic membrane	*PDLIM4/ANK3/GRIK4/GRIK1/CBLN1/TANC1/CHRM2/ABI1/GABRA5/SSPN/GABRG1/GRM7/NETO1/GABRB3*	*p* < 0.0001
GO:0098794	postsynapse	*ADD2/PDLIM4/ANK3/GRIK4/GRIK1/CBLN1/CALB1/TANC1/CHRM2/ABI1/GABRA5/SSPN/GABRG1/GRM7/NETO1/CDH2/KCNN2/GABRB3*	*p* = 0.0004
GO:0097060	synaptic membrane	*PDLIM4/ANK3/GRIK4/GRIK1/CBLN1/TANC1/CHRM2/ABI1/GABRA5/SSPN/GABRG1/GRM7/NETO1/GABRB3*	*p* = 0.0006
MF			
GO:0005338	nucleotide-sugar transmembrane transporter activity	*SLC35D2/SLC35C1/SLC35A1*	*p* = 0.0005

GO, gene ontology; BP, biological process; CC, cellular component; MF, molecular function.

### Replication Stage

In the replication stage, 5 of 10 CpG sites in 8 candidate genes from 850K BeadChip results were verified to be statistically different (*p* < 0.05). The trend of the methylation level of the rest 5 CpG sites with no significant differences is consistent with the 850k BeadChip results (Additional File S3), suggesting that the 850k results are reliable. The genes screened by GO analysis are worthy of further verification.

In 188 paired samples, 203 of 308 CpG sites from 17 CpG islands in the promoter regions of 8 candidate genes were significantly different (*p* < 0.05) between the IS and control group (Additional File S4). Consistent with the 850k BeadChip results, the methylation level of all 203 CpG sites was lower in the IS group than in the control group. 13 of 17 CpG islands were differentially methylated between IS patients and controls (*p* < 0.05, Additional File S5). 7 genes (*CDH2/PCDHB10/PCDHB11/PCDHB14/PCDHB16/PCDHB3/PCDHB9*) showed differences (*p* < 0.05) between the two groups even if adjusting for smoking, drinking, history of hypertension and diabetes, and blood lipid levels, and adjust FDR *p* values of 6 genes (*CDH2/PCDHB10/PCDHB11/PCDHB16/PCDHB3/PCDHB9*) were statistically significant (*p* < 0.05, Table 3). The methylation levels of the 7 genes were highly correlated in both the case group and the control group (*p* < 0.05, Additional File S6). Besides, we further split the samples by sex (male or female). There were 7 differentially methylated genes (*CDH2/PCDHB10/PCDHB11/PCDHB14/PCDHB16/PCDHB3/PCDHB9*) in the male subgroup and 4 (*CDH2/PCDHB10/PCDHB11/PCDHB9*) in the female subgroup, which is shown in Table 4.

**TABLE 3:** Validation of 8 candidate genes in the ischemic stroke group and control group.

Target	Group difference	*p* value	Adjust *p* value	Adjust FDR *p* value
*CDH2*	−0.0127	*p* < 0.0001*	*p* < 0.0001*	*p* = 0.0001*
*PCDHB10*	−0.0228	*p* < 0.0001*	*p* < 0.0001*	*p* = 0.0002*
*PCDHB11*	−0.0232	*p* < 0.0001*	*p* = 0.0001*	*p* = 0.0006*
*PCDHB14*	−0.0193	*p* = 0.0002*	*p* = 0.0498*	*p* = 0.0940
*PCDHB16*	−0.0162	*p* = 0.0117*	*p* = 0.0148*	*p* = 0.0315*
*PCDHB3*	−0.0158	*p* = 0.0016*	*p* = 0.0089*	*p* = 0.0217*
*PCDHB6*	−0.0087	*p* = 0.3376	*p* = 0.3399	*p* = 0.5268
*PCDHB9*	−0.0251	*p* < 0.0001*	*p* = 0.0002*	*p* = 0.0008*

Adjusted factors: smoking, drinking, previous medical history of hypertension and diabetes mellitus, and plasma lipid levels (total triglyceride, total cholesterol, high-density lipoprotein, and low-density lipoprotein). *Statistically significant difference (p < 0.05).

**TABLE 4:** Stratified analysis by gender.

Target	Group difference	*p* value	Adjust *p* value
Male (case:control = 144:144)			
*CDH2*	−0.0118	*p* < 0.0001*	*p* = 0.0006*
*PCDHB10*	−0.0211	*p* = 0.0001*	*p* < 0.0001*
*PCDHB11*	−0.0216	*p* = 0.0005*	*p* = 0.0003*
*PCDHB14*	−0.0201	*p* = 0.0002*	*p* = 0.0139*
*PCDHB16*	−0.0153	*p* = 0.0327*	*p* = 0.0070*
*PCDHB3*	−0.0139	*p* = 0.0081*	*p* = 0.0038*
*PCDHB6*	−0.0038	*p* = 0.7111	*p* = 0.3113
*PCDHB9*	−0.0230	*p* = 0.0003*	*p* = 0.0003*
Female (case:control = 44:44)			
*CDH2*	−0.0158	*p* = 0.0014*	*p* = 0.0299*
*PCDHB10*	−0.0283	*p* = 0.0103*	*p* = 0.0270*
*PCDHB11*	−0.0283	*p* = 0.0129*	*p* = 0.0160*
*PCDHB14*	−0.0164	*p* = 0.2108	*p* = 0.0562
*PCDHB16*	−0.0192	*p* = 0.1810	*p* = 0.2095
*PCDHB3*	−0.0220	*p* = 0.0875	*p* = 0.0907
*PCDHB6*	−0.0247	*p* = 0.2093	*p* = 0.3205
*PCDHB9*	−0.0317	*p* = 0.0124*	*p* = 0.0195*

Adjusted factors: smoking, drinking, previous medical history of hypertension and diabetes mellitus, and plasma lipid levels (total triglyceride, total cholesterol, high-density lipoprotein, and low-density lipoprotein). *Statistically significant difference (p < 0.05).

### Stratified Analysis by IS Subtypes and Location

We split the case samples by IS subtypes (TOAST classification), stratified by LAA (*n* = 67), CE (*n* = 13), SVD (*n* = 64), ODC (*n* = 5), and UND (*n* = 39). However, no analysis was conducted in the CE and ODC groups because of the small sample size. We also did not perform analysis in the UND group as it is hard to identify the cause of IS. There were 8 differentially methylated genes (*CDH2/PCDHB10/PCDHB11/PCDHB14/PCDHB16/PCDHB3/PCDHB6/PCDHB9*) in the LAA subgroup and 1 (*CDH2*) in the SVD subgroup after adjusting for smoking, drinking, history of hypertension and diabetes, and blood lipid levels (*p* < 0.05, Table 5). Besides, the case samples were seperated according to the location of the lesion (anterior circulation-left hemisphere, *n* = 72; anterior circulation-right hemisphere, *n* = 63; posterior circulation, *n* = 48; and both anterior and posterior circulation, *n* = 5). We also did not perform analysis in the “both anterior and posterior circulation” group because of the small sample size. Four genes (*CDH2/PCDHB10/PCDHB11/PCDHB9*) in the anterior circulation-left hemisphere subgroup, 8 genes (*CDH2/PCDHB10/PCDHB11/PCDHB14/PCDHB16/PCDHB3/PCDHB6/PCDHB9*) in the anterior circulation-right hemisphere subgroup and 4 genes (*CDH2/PCDHB10/PCDHB11/PCDHB9*) in the posterior circulation subgroup showed differences after adjusting for smoking, drinking, history of hypertension and diabetes, and blood lipid levels (*p* < 0.05, Table 6).

**TABLE 5:** Stratified analysis by ischemic stroke subtypes.

Target	Group difference	*p* value	Adjust *p* value
LAA (case:control = 67:67)			
*CDH2*	−0.0138	*p* = 0.0016*	*p* = 0.0059*
*PCDHB10*	−0.0249	*p* = 0.0015*	*p* = 0.0028*
*PCDHB11*	−0.0264	*p* = 0.0035*	*p* = 0.0034*
*PCDHB14*	−0.0273	*p* = 0.0016*	*p* = 0.0070*
*PCDHB16*	−0.0204	*p* = 0.0597	*p* = 0.0043*
*PCDHB3*	−0.0182	*p* = 0.0247*	*p* = 0.0035*
*PCDHB6*	−0.0146	*p* = 0.3849	*p* = 0.0434*
*PCDHB9*	−0.0292	*p* = 0.0014*	*p* = 0.0015*
SVD (case:control = 64:64)			
*CDH2*	−0.0101	*p* = 0.0022*	*p* = 0.0343*
*PCDHB10*	−0.0190	*p* = 0.0322*	*p* = 0.1069
*PCDHB11*	−0.0195	*p* = 0.0502	*p* = 0.1195
*PCDHB14*	−0.0131	*p* = 0.1816	*p* = 0.3445
*PCDHB16*	−0.0151	*p* = 0.1758	*p* = 0.3626
*PCDHB3*	−0.0093	*p* = 0.3182	*p* = 0.5970
*PCDHB6*	−0.0031	*p* = 0.8273	*p* = 0.9188
*PCDHB9*	−0.0117	*p* = 0.2385	*p* = 0.4120

Adjusted factors: smoking, drinking, previous medical history of hypertension and diabetes mellitus, and plasma lipid levels (total triglyceride, total cholesterol, high-density lipoprotein, and low-density lipoprotein). *Statistically significant difference (p < 0.05). LAA, large-artery atherosclerosis; SVD, small vessel disease.

**TABLE 6:** Stratified analysis by ischemic stroke location.

Target	Group difference	*p* value	Adjust *p* value
Anterior circulation-left hemisphere (case:control = 72:72)			
*CDH2*	−0.0133	*p* = 0.0005*	*p* = 0.0015*
*PCDHB10*	−0.0200	*p* = 0.0201*	*p* = 0.0278*
*PCDHB11*	−0.0191	*p* = 0.0419*	*p* = 0.0400*
*PCDHB14*	−0.0155	*p* = 0.0997	*p* = 0.2873
*PCDHB16*	−0.0111	*p* = 0.2982	*p* = 0.1031
*PCDHB3*	−0.0093	*p* = 0.3007	*p* = 0.1201
*PCDHB6*	0.0034	*p* = 0.8128	*p* = 0.3847
*PCDHB9*	−0.0237	*p* = 0.0214*	*p* = 0.0144*
Anterior circulation-right hemisphere (case:control = 63:63)			
*CDH2*	−0.0103	*p* = 0.0005*	*p* = 0.0058*
*PCDHB10*	−0.0249	*p* = 0.0026*	*p* = 0.0037*
*PCDHB11*	−0.0257	*p* = 0.0075*	*p* = 0.0060*
*PCDHB14*	−0.0219	*p* = 0.0067*	*p* = 0.0470*
*PCDHB16*	−0.0204	*p* = 0.0644	*p* = 0.0243*
*PCDHB3*	−0.0224	*p* = 0.0051*	*p* = 0.0076*
*PCDHB6*	−0.0248	*p* = 0.1181	*p* = 0.0276*
*PCDHB9*	−0.0257	*p* = 0.0052*	*p* = 0.0090*
Posterior circulation (case:control = 48:48)			
*CDH2*	−0.0174	*p* = 0.0022*	*p* = 0.0363*
*PCDHB10*	−0.0243	*p* = 0.0066*	*p* = 0.0113*
*PCDHB11*	−0.0265	*p* = 0.0064*	*p* = 0.0196*
*PCDHB14*	−0.0187	*p* = 0.0557	*p* = 0.0717
*PCDHB16*	−0.0190	*p* = 0.1315	*p* = 0.1532
*PCDHB3*	−0.0172	*p* = 0.0661	*p* = 0.1380
*PCDHB6*	−0.0112	*p* = 0.5626	*p* = 0.9945
*PCDHB9*	−0.0268	*p* = 0.0075*	*p* = 0.0205*

Adjusted factors: smoking, drinking, previous medical history of hypertension and diabetes mellitus, and plasma lipid levels (total triglyceride, total cholesterol, high-density lipoprotein, and low-density lipoprotein). *Statistically significant difference (p < 0.05).

### The Association Between DNA Methylation in 7 Differentially Methylated Genes and IS

The methylation level of each gene was grouped into quartiles. The DNA methylation level of the 7 differentially methylated genes in Q4 groups was associated with higher risk of IS than Q1 groups (*p* < 0.05), which are listed in Table 7.

**TABLE 7:** Associations between DNA methylation in 7 differentially methylated genes and risk of ischemic stroke.

Target	OR	95%CI	*p* value	Adjust *p* value
*CDH2*				
Q2 vs. Q1	8.844	3.776–20.710	*p* < 0.0001*	*p* < 0.0001*
Q3 vs. Q1	6.092	2.593–14.309	*p* < 0.0001*	*p* < 0.0001*
Q4 vs. Q1	24.615	9.518–63.660	*p* < 0.0001*	*p* < 0.0001*
*PCDHB10*				
Q2 vs. Q1	2.634	1.363–5.089	*p* = 0.0040*	*p* = 0.0004*
Q3 vs. Q1	3.627	1.775–7.413	*p* = 0.0004*	*p* = 0.0001*
Q4 vs. Q1	7.797	3.707–16.399	*p* < 0.0001*	*p* < 0.0001*
*PCDHB11*				
Q2 vs. Q1	2.694	1.371–5.296	*p* = 0.0040*	*p* = 0.0016*
Q3 vs. Q1	4.103	1.982–8.497	*p* = 0.0001*	*p* = 0.0003*
Q4 vs. Q1	6.346	2.963–13.592	*p* < 0.0001*	*p* < 0.0001*
*PCDHB14*				
Q2 vs. Q1	1.402	0.774–2.540	*p* = 0.2653	*p* = 0.3518
Q3 vs. Q1	4.035	2.051–7.937	*p* < 0.0001*	*p* = 0.0055*
Q4 vs. Q1	4.048	2.053–7.983	*p* < 0.0001*	*p* = 0.0028*
*PCDHB16*				
Q2 vs. Q1	2.862	1.537–5.327	*p* = 0.0009*	*p* = 0.0007*
Q3 vs. Q1	1.319	0.671–2.595	*p* = 0.4221	*p* = 0.2949
Q4 vs. Q1	3.355	1.743–6.456	*p* = 0.0003*	*p* = 0.0002*
*PCDHB3*				
Q2 vs. Q1	1.390	0.764–2.529	*p* = 0.2813	*p* = 0.0897
Q3 vs. Q1	2.032	1.125–3.671	*p* = 0.0187*	*p* = 0.0055*
Q4 vs. Q1	2.965	1.561–5.630	*p* = 0.0009*	*p* = 0.0016*
*PCDHB9*				
Q2 vs. Q1	2.402	1.221–4.725	*p* = 0.0111*	*p* = 0.0008*
Q3 vs. Q1	3.500	1.799–6.807	*p* = 0.0002*	*p* = 0.0010*
Q4 vs. Q1	5.336	2.656–10.720	*p* < 0.0001*	*p* < 0.0001*

Adjusted factors: smoking, drinking, previous medical history of hypertension and diabetes mellitus, and plasma lipid levels (total triglyceride, total cholesterol, high-density lipoprotein, and low-density lipoprotein). *Statistically significant difference (p < 0.05). OR odds ratio; CI, confidence interval.

### Biomarker Potential of the 7 Differentially Methylated Genes for IS

**FIGURE 4:**
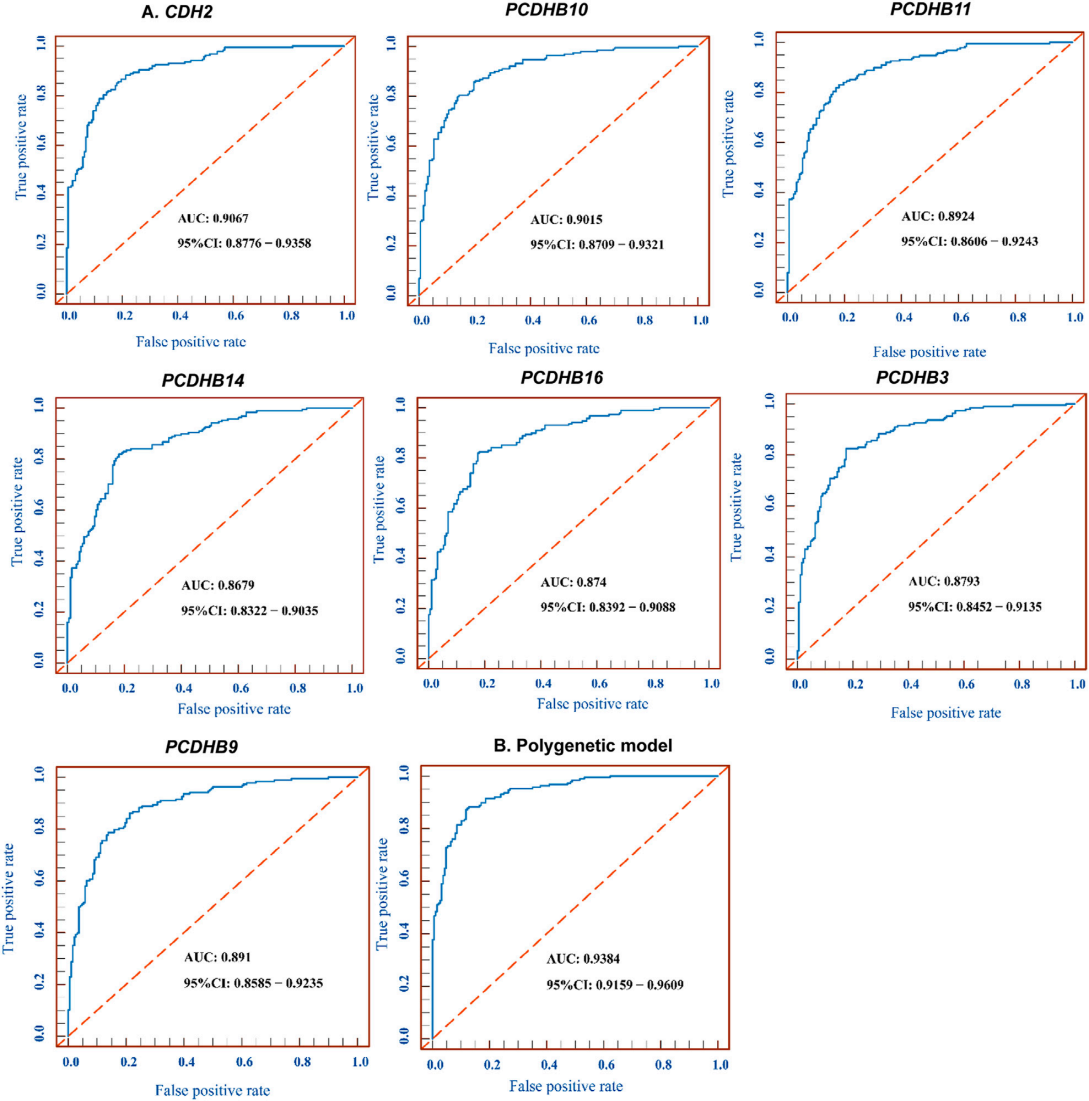
ROC for 7 differentially methylated genes and polygenic methylation model. **(A)** ROC curve measuring sensitivity (*Y* axis) and 1-specificity (*X* axis) of methylation of *CDH2/PCDHB10/PCDHB11/PCDHB14/PCDHB16/PCDHB3/PCDHB9* genes as biomarkers for discriminating IS from controls. **(B)** ROC curve of the polygenic methylation model. AUC, area under the curve; ROC, receiver operating characteristic; CI, confidence interval.

We tested the biomarker potential of the 7 differentially methylated genes using receiver operating characteristic (ROC) analysis. The sensitivity, specificity, accuracy and area under the curve (AUC) in *CDH2/PCDHB10/PCDHB11/PCDHB14/PCDHB16/PCDHB3/PCDHB9* genes was determined based on methylation values of replication stage (Table 8, Figure 4A). To enhance the diagnostic ability of these differentially methylated genes in IS, we tested the performance of the polygenic methylation model. The sensitivity, specificity, accuracy and AUC of polygenic methylation model was shown in Table 8 and Figure 4B using ROC analysis. The polygenic methylation model performed better than each of the individual genes, especially with higher specificity (0.8723), sensitivity (0.883), accuracy (0.8777), and AUC (0.9384).

**TABLE 8:** ROC analysis of 7 differentially methylated genes and polygenic methylation model.

Target	Adjust AUC	Adjust Sensitivity	Adjust Specificity	Adjust Accuracy
*CDH2*	0.9067	0.883	0.7872	0.8351
*PCDHB10*	0.9015	0.8617	0.7979	0.8298
*PCDHB11*	0.8924	0.8298	0.8191	0.8245
*PCDHB14*	0.8679	0.8191	0.8191	0.8191
*PCDHB16*	0.874	0.8245	0.8191	0.8218
*PCDHB3*	0.8793	0.8245	0.8245	0.8245
*PCDHB9*	0.891	0.8617	0.7872	0.8245
Polygenic model	0.9384	0.883	0.8723	0.8777

Adjusted factors: smoking, drinking, previous medical history of hypertension and diabetes mellitus, and plasma lipid levels (total triglyceride, total cholesterol, high-density lipoprotein, and low-density lipoprotein). AUC area under the curve; ROC receiver operating characteristic.

### Validation of the mRNA Expression of Differentially Methylated Genes

**FIGURE 5:**
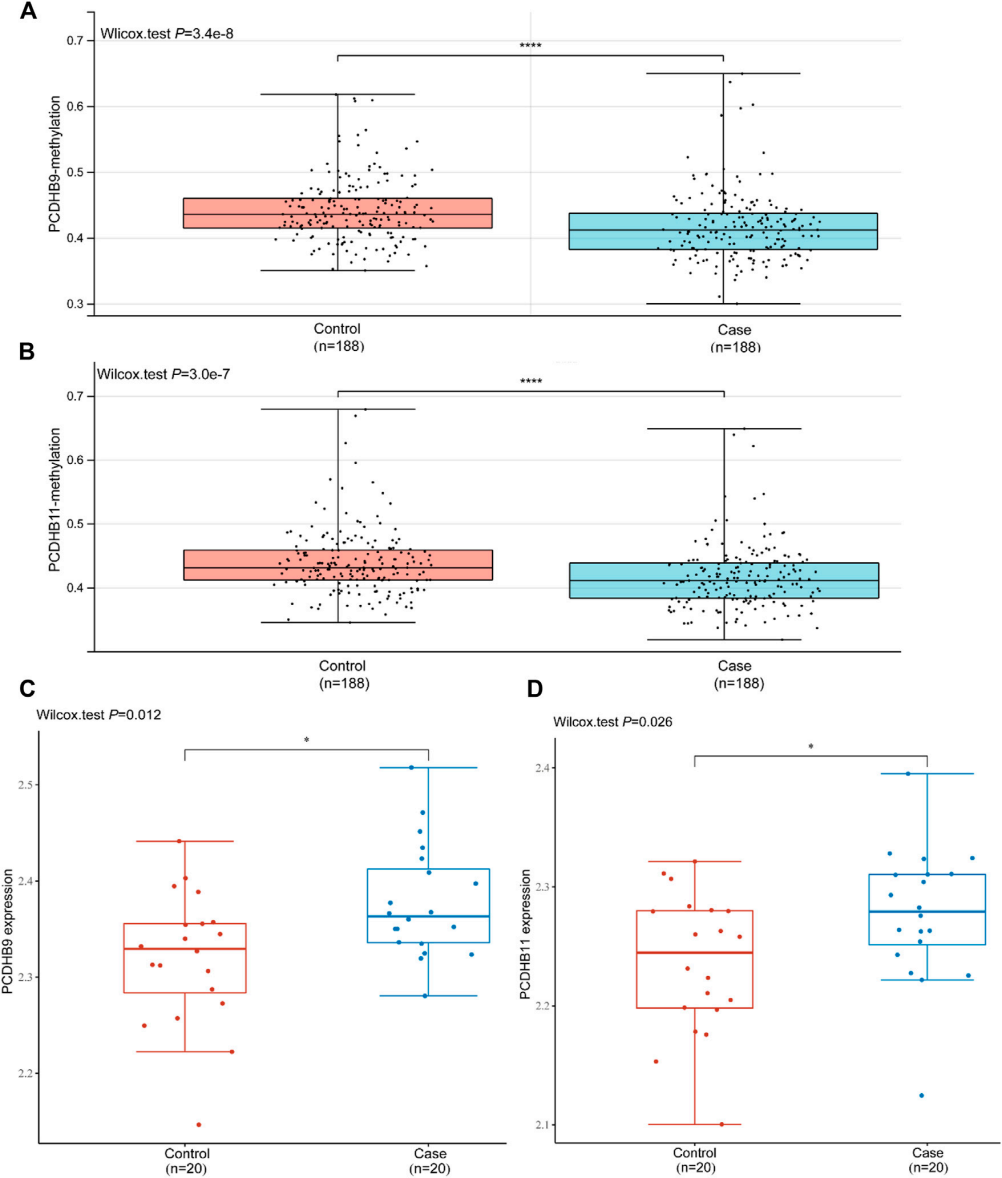
Validation of the mRNA expression of *PCDHB9* and *PCDHB11* in GSE22255. **(A)**: Methylation level of the *PCDHB9* gene in the control and case (IS) groups; **(B)**: Methylation level of the *PCDHB11* gene in the control and case (IS) groups; **(C)**: mRNA expression of the *PCDHB9* gene in the control and case (IS) groups; **(D)**: mRNA expression of the *PCDHB11* gene in the control and case (IS) groups. *Statistically significant difference (*p* < 0.05). ****Statistically significant difference (*p* < 0.0001).

In the replication stage, there were 7 differentially hypomethylated genes (*CDH2/PCDHB10/PCDHB11/PCDHB14/PCDHB16/PCDHB3/PCDHB9*) in the IS group. Generally, a decrease in DNA methylation leads to abnormal gene transcription, resulting in the upregulation of gene expression. We validated the expression levels of these 7 genes in the GSE22255 dataset. The expression of two genes (*PCDHB9* and *PCDHB11*) increased, while the methylation level of the same 2 genes decreased in IS patients compared to controls (*p* < 0.05, Figure 5). The expression levels of the other three genes (*CDH2/PCDHB14/PCDHB16*) also increased in the IS group, but there were no significant differences between the two groups (*p* > 0.05, Additional Files S7–11).

## Discussion

Certainly, IS is a heterogeneous disease with a genetic predisposition (Acosta et al., 2021). Emerging evidence has revealed the importance of epigenetic regulations in IS, particularly DNA methylation (Gómez-Úriz et al., 2015; Davis Armstrong et al., 2018; Shen et al., 2019; Soriano-Tárraga et al., 2020). Our study presented genome-wide alterations in DNA methylation in IS patients and controls by identifying a total of 462 functional DMPs corresponding to 373 annotated genes and revealed that hypomethylated sites were eightfold more numerous than DNA hypermethylation sites in IS cases, demonstrating that the hypomethylated modification was predominant in IS.

For DNA methylation profiling, GO analysis showed that these genes form interconnected networks involved in the calcium-dependent cell-cell adhesion via plasma membrane cell adhesion molecules, homophilic cell adhesion via plasma membrane adhesion molecules, cell-cell adhesion via plasma-membrane adhesion molecules and other networks. Among them, the most enriched term in GO analysis was “calcium-dependent cell-cell adhesion via plasma membrane cell adhesion molecules” item including 8 candidate genes (*CDH2/PCDHB10/PCDHB11/PCDHB14/PCDHB16/PCDHB3/PCDHB6/PCDHB9*), which were related to the cadherins.

Cadherins are a superfamily of calcium-dependent adhesion molecules mainly involved in tissue and embryonic cell development (Schaarschuch and Hertel, 2018). They can be classified into several subfamilies such as classic cadherins (CDHs), protocadherins (PCDHs), desmosomal cadherins and so on (Pancho et al., 2020). The *PCDH* gene family comprises three gene clusters (*PCDHA, PCDHB*, and *PCDHG*, respectively) (Peek et al., 2017; Canzio and Maniatis, 2019). PCDHs are highly expressed in the central nervous system in neurons, astrocytes, pericytes and brain microvascular endothelial cells, and mediate various developmental processes, including synaptic maintenance, neuronal survival, and spatial patterning of axons and dendrites (Gabbert et al., 2020; Mancini et al., 2020; Ing-Esteves et al., 2018). PCDHs have been reported to be relevant to neurodegenerative diseases, epilepsy, schizophrenia and mood disorders (Schaarschuch and Hertel, 2018; Langfelder et al., 2016; Li et al., 2017; Pederick et al., 2018; Lencz et al., 2021; Flaherty and Maniatis, 2020). However, the association between PCDHs and IS is rarely reported. Ana et al. found altered *PCDHGA3* gene expression was strongly associated with reduced stroke volume (Ortega et al., 2016). Some studies revealed that cadherins are involved in the regulation of angiogenesis and inflammation in endothelial cells and lack of PCDHs may lead to the destruction of the blood-brain barrier (Gabbert et al., 2020; Nanes et al., 2012), which are the mechanisms of IS. However, no direct evidence of the association between *PCDHB* genes and IS has been shown in previous studies. The semi-stochastic expression of *PCDH* genes is regulated by DNA methylation (Hirayama and Yagi, 2017). In our results, the methylation level of *PCDHB (PCDHB10/PCDHB11/PCDHB14/PCDHB16/PCDHB3/PCDHB9*) genes was lower in IS cases compared with controls, which suggested the methylation status of *PCDHB* genes may be involved in the pathogenesis of IS and be potential diagnostic biomarkers for IS.

Like the PCDHs, classic CDHs have been implicated in neurulation, brain development, and regulation of synaptic function (Polanco et al., 2021; de Agustín-Durán et al., 2021; Sanes and Zipursky, 2020). They are also involved in the formation of atherosclerotic plaques (He et al., 2017), which is an important clinical feature of IS. One of the most important members of CDHs is the neuronal cadherin (N-cadherin, *CDH2*) (Schaarschuch and Hertel, 2018; Martinez-Garay, 2020). So far, there has been no direct evidence to show the relationship between *CDH2* gene and stroke. A Study about myocardial infarction revealed overexpression of *CDH2*, *CDH12*, *PCDH17*, and *PCDH18* in myocardial infarction vascular smooth muscle cells compared with controls (Derda et al., 2018). In this study we found decreased methylation level of the promoter region in *CDH2* gene. Generally, hypomethylation in promotor is believed to upregulate gene transcription, which is consistent with the previous study of myocardial infarction in a way. This is the first time we have found the correlation between the methylation of *CDH2* gene and IS.

There are also some interesting findings in our study. First, when we split the samples by sex, we found fewer differentially methylated genes (4 genes) in females, which we thought might be because the sample size of women is much smaller than that of men. What’s more, compared with SVD-control groups, there are more differentially methylated genes in LAA-control groups. We thought it might hint that the LAA subtype may be more susceptible to epigenetic regulation, and further studies are needed. Lastly, there existed more differentially methylated genes in the right hemisphere-control groups than the other two paired groups, which need further studies in a large sample size.

The peripheral blood is a good choice for epigenetic research of IS, as it is easy to obtain with minor invasion (Qin et al., 2019). Moreover, IS is a disease related to the vasculature and interrupting blood supply to the brain. Liu et al. found that stroke patients had lower methylation level of the *ACTB* gene in blood (Liu et al., 2021), which revealed that peripheral blood could identify the methylation aberrations associated with stroke.

There were certain limitations in our study. First, gene expression was verified in a public dataset, but not explored in our blood samples. Our study aimed to describe the global DNA methylation patterns in IS and to explore potential diagnostic biomarkers for IS in a Chinese population. Although gene and protein expression was not the main purpose of our research, it will be carried out in follow-up studies. In addition, we couldn’t detect the methylation level of more CpG sites due to the limited funds. However, they will be the directions for our future study.

## Conclusion

The present study demonstrated the changes in genome-wide DNA methylation between IS cases and controls and identified 7 novel DNA methylation genes (*CDH2/PCDHB10/PCDHB11/PCDHB14/PCDHB16/PCDHB3/PCDHB9*) related to IS in the replication stage. These data may provide preliminary evidence for further exploring the role of DNA methylation in IS.

## Data Availability

The datasets presented in this article are not readily available because of ethical restrictions. Requests to access the datasets should be directed to the corresponding authors.

## Abbreviations

CDHs, cadherins; CE, cardioembolism; CpG, cytosine phosphate guanine; DMPs, DNA methylation positions; GO, gene ontology; HDL, high-density lipoprotein; IS, ischemic stroke; KEGG, Kyoto Encyclopedia of Genes and Genomes; LAA, large-artery atherosclerosis; LDL, low-density lipoprotein; ODC, other determined cause; PCDHs, protocadherins; ROC, receiver operating characteristic; SVD, small vessel disease; TC, total cholesterol; TG, total triglyceride; TOAST, Trial of ORG 10172 in Acute Stroke Treatment; UND, undetermined; 5′UTR, 5′untranslated region; AUC, area under the curve.
