# Supplementary material for: Association of DNA Methylation Patterns in 7 Novel Genes With Ischemic Stroke in the Northern Chinese Population
Source: Front Genet. 2022 Apr 11;13:844141. doi: 10.3389/fgene.2022.844141 (PMC9035884; doi:10.3389/fgene.2022.844141)
Supplement: Supplementary file 2 [file DataSheet2.PDF]

Additional file 2

462 functional DMPs

probe	CHR	MAPINFO	Gene Name	UCSC_Ref Gene_Group_Anovar
cg13503915	19	52134227	*SIGLEC5*	upstream
cg14011486	1	26737247	*LIN28*	upstream
cg15577010	2	232395273	*NMUR1*	upstream
cg06571939	22	50701233	*MAPK12*	upstream;downstream
cg19925512	22	32750881	*RFPL3*	UTR5
cg16658020	17	39203641	*KRTAP2-1*	upstream
cg18188350	7	57207975	*ZNF479*	upstream
cg18856296	11	60609242	*CCDC86*	upstream
cg16333262	14	76617755	*C14orf118*	upstream
cg16166011	2	70996021	*ADD2*	upstream
cg18758796	5	131593413	*PDLIM4*	UTR5
cg04927537	17	76976091	*LGALS3BP*	upstream
cg26086917	16	89679694	*DPEP1*	upstream
cg19477247	3	113955994	*ZNF80*	UTR5
cg17707165	15	98103935	*LOC101927310*	upstream
cg00152799	18	54306261	*TXNL1*	upstream
cg07378309	3	121312164	*FBXO40*	upstream
cg20512303	5	131592959	*PDLIM4*	upstream
cg10454162	3	113955940	*ZNF80*	UTR5
cg03981535	1	201951234	*RNPEP*	upstream
cg02518129	21	27946082	*CYYR1*	upstream
cg03378815	18	28623390	*DSC3*	upstream
cg12564285	5	131593104	*PDLIM4*	upstream
cg11945372	22	18507747	*MICAL3*	upstream
cg02571142	8	42234803	*DKK4*	upstream
cg16340422	17	17110120	*PLD6*	upstream
cg08691338	16	84732980	*USP10*	upstream
cg16288713	13	79234144	*RNF219*	upstream
cg25983544	18	23713626	*PSMA8*	upstream
cg19871388	20	50419082	*SALL4*	upstream
cg01312482	5	178451176	*ZNF879*	UTR5
cg17344060	1	94345166	*DNTTIP2*	upstream
cg09190290	16	71927972	*IST1*	upstream
cg00191629	13	110439004	*IRS2*	upstream
cg00162304	11	60282281	*MS4A13*	upstream
cg27228211	7	27778939	*TAX1BP1*	upstream

cg08546933	7	79763240	*GNAI1*	upstream
cg18634211	1	26737262	*LIN28*	UTR5
cg09547190	9	95857577	*C9orf89*	upstream
cg08458252	13	46961871	*C13orf18*	UTR5
cg11008866	3	121902442	*CASR*	upstream
cg23940614	15	84115479	*SH3GL3*	upstream
cg19727801	5	140619446	*PCDHB19P*	upstream
cg25108548	12	13196954	*KIAA1467*	upstream
cg16673296	20	30017604	*DEFB122*	upstream
cg18951352	6	149806077	*ZC3H12D*	UTR5
cg26075090	4	25865375	*SEL1L3*	upstream
cg24027965	2	149894786	*LYPD6B*	upstream
cg18016034	10	61900573	*ANK3*	UTR5
cg25178683	17	76976267	*LGALS3BP*	upstream
cg05938007	2	27994018	*MRPL33*	upstream
cg03829839	12	58146821	*CDK4*	upstream
cg07150062	14	104552032	*ASPG*	UTR5
cg24624629	17	39203621	*KRTAP2-1*	upstream
cg02415057	12	75784884	*GLIPR1L2*	UTR5
cg07348641	13	25746495	*FAM123A*	upstream;downstream
cg23312397	14	96670738	*BDKRB2(*	upstream
cg21117330	5	140772286	*PCDHGA4*	UTR5
cg00878163	17	56565644	*HSF5*	UTR5
cg11595575	5	140235510	*PCDHA6*	UTR5
cg15951740	12	46660255	*SLC38A1*	UTR5
cg02726377	4	108745279	*SGMS2*	upstream
cg16672337	19	22605111	*ZNF98*	UTR5
cg12351126	12	75784864	*GLIPR1L2*	UTR5
cg17885404	8	67976175	*CSPP1*	upstream
cg04862679	11	59578540	*MRPL16*	upstream
cg03945604	19	40724609	*TTC9B*	upstream
cg14338032	20	2081880	*STK35*	upstream
cg03350299	2	21266960	*APOB*	upstream
cg10982664	5	78407418	*BHMT*	upstream
cg08198187	12	31882596	*AMN1*	upstream
cg22084258	11	120435389	*GRIK4*	UTR5
cg16134349	10	61900552	*ANK3*	UTR5
cg26023019	21	31311859	*GRIK1*	UTR5
cg03389653	16	49316197	*CBLN1*	upstream
cg00108944	12	75784855	*GLIPR1L2*	UTR5
cg25154586	4	175444627	*HPGD*	upstream
cg07301105	2	232379665	*C2orf52*	upstream

cg08834401	2	71017846	*FIGLA*	upstream
cg03578013	3	24145718	*LOC152024*	upstream
cg23062385	6	143928702	*PHACTR2*	upstream
cg14663451	8	32504919	*NRG1*	UTR5
cg15834814	19	9609704	*ZNF560*	upstream
cg06743973	16	28985149	*SPNS1*	upstream
cg06484167	6	27832180	*HIST1H2AL*	upstream
cg07814122	12	13196942	*KIAA1467*	upstream
cg08168630	11	4390117	*OR52B4*	upstream
cg14015441	8	105479318	*DPYS*	upstream
cg04172000	19	16771088	*TMEM38A*	upstream
cg02320481	13	110438578	*IRS2*	UTR5
cg10368049	7	94023796	*COL1A2*	upstream
cg00711090	19	57018614	*ZNF471*	upstream
cg19836999	16	68270498	*ESRP2*	upstream
cg02986414	12	101188350	*ANO4*	upstream
cg21522026	5	140602959	*PCDHB14*	UTR5
cg09704056	1	98516154	*MIR137HG*	upstream
cg21215899	5	121414425	*LOX*	upstream
cg13922962	6	169654390	*THBS2*	upstream
cg14533206	13	110439006	*IRS2*	upstream
cg20457147	14	61787823	*PRKCH*	upstream
cg25162921	9	21801897	*MTAP*	upstream
cg10175387	9	138853745	*UBAC1*	upstream
cg07630707	12	132194958	*SFSWAP*	upstream
cg06870925	19	58962383	*ZNF324B*	upstream
cg07325776	8	91095455	*CALB1*	upstream
cg17090409	11	74443005	*CHRDL2*	upstream
cg22487198	13	48611020	*NUDT15*	upstream
cg02605776	1	111507160	*C1orf103*	upstream
cg03764335	1	248568657	*OR2T1*	upstream
cg17110239	3	62860721	*CADPS*	UTR5
cg23839761	9	96108838	*C9orf129*	upstream
cg04579398	19	36485360	*SDHAF1*	upstream
cg23140290	13	26797279	*RNF6*	upstream
cg12302402	11	18230629	*LOC494141*	upstream
cg06961147	2	159824263	*TANC1*	upstream
cg04996219	5	11904110	*CTNND2*	UTR5
cg21068090	4	52917303	*SPATA18*	upstream
cg06808011	16	3286070	*ZNF200*	upstream
cg02866106	7	136553110	*CHRM2*	upstream
cg06259125	6	168720679	*DACT2*	upstream

cg06961429	14	101493361	*MIR329-2*	upstream;downstream
cg03612569	15	48483440	*CTXN2*	upstream
cg00668034	1	28098848	*STX12*	upstream
cg07909285	8	106330885	*ZFPM2*	upstream
cg17501395	6	149806331	*ZC3H12D*	upstream
cg13891189	9	116164001	*ALAD*	upstream
cg16878332	2	84743573	*DNAH6*	upstream
cg19507068	7	79764176	*GNAI1*	UTR5
cg20480368	3	174576768	*NAALADL2*	upstream
cg03000419	11	82997694	*CCDC90B*	upstream
cg02961664	3	81811363	*GBE1*	upstream
cg15235893	2	235406363	*ARL4C*	upstream
cg24197374	5	140800686	*PCDHGA11*	UTR5
cg18242139	1	50574837	*ELAVL4*	UTR5
cg21337580	8	11141424	*MTMR9*	upstream
cg00510507	10	61900413	*ANK3*	UTR5
cg21160842	11	32112091	*RCN1*	upstream
cg22033530	19	55692003	*SYT5*	upstream;downstream
cg10761315	14	104552034	*ASPG*	UTR5
cg04518342	5	131593106	*PDLIM4*	upstream
cg07168818	9	135285198	*C9orf171*	upstream
cg22550299	6	169654842	*THBS2*	upstream
cg03084288	19	4277656	*SHD*	upstream
cg18663063	5	68710907	*MARVELD2*	upstream;downstream
cg02233750	22	43539658	*MCAT*	upstream
cg18009000	10	71811927	*H2AFY2*	upstream
cg23358625	19	23945641	*RPSAP58*	upstream
cg16648019	18	53253316	*TCF4*	UTR5
cg24018665	12	108522859	*WSCD2*	upstream
cg26963093	2	88469730	*THNSL2*	upstream
cg03033508	13	26797157	*RNF6*	upstream
cg20106459	19	55866087	*COX6B2*	UTR5
cg06916670	1	202829945	*LOC148709*	upstream
cg25010500	5	140619586	*PCDHB19P*	upstream
cg06584561	13	31456352	*LINC00545*	upstream;downstream
cg01216565	17	80607047	*WDR45B*	upstream
cg06209491	1	40138402	*NT5C1A*	upstream
cg08057103	13	110438916	*IRS2*	upstream
cg08357436	17	56565641	*HSF5*	UTR5
cg21964800	11	63272764	*LGALS12*	upstream
cg01020079	12	51236704	*TMPRSS12*	UTR5
cg10767045	10	89264128	*MINPP1*	upstream;downstream

cg13002243	14	27291831	*LOC101927062*	upstream
cg06960024	10	27150529	*ABI1*	upstream
cg20104432	7	138817981	*TTC26*	upstream
cg12463140	19	17665823	*COLGALT1*	upstream
cg07688291	7	123431142	*RNU6-2*	upstream
cg11630226	8	143781816	*LY6K*	UTR5
cg22034203	1	31231421	*LAPTM5*	upstream
cg11235829	1	44439777	*ATP6V0B*	upstream;downstream
cg06483432	4	55523233	*KIT*	upstream
cg14387743	6	133056278	*VNN3*	upstream
cg14630685	12	57630931	*NDUFA4L2*	UTR5
cg03698781	1	35135423	*MIR552*	upstream
cg13405783	8	54164296	*OPRK1*	upstream
cg01892167	11	4208212	*LOC100506082*	upstream
cg16229875	14	23589066	*CEBPE*	upstream
cg26803268	10	18549536	*CACNB2*	UTR5
cg07163456	3	192636424	*C3orf59*	upstream
cg15054400	1	94345486	*DNTTIP2*	upstream
cg22490229	1	248342534	*OR2M2*	upstream
cg03760072	3	133502685	*SRPRB*	upstream
cg14013591	20	57874597	*EDN3*	upstream
cg15564579	3	119298083	*ADPRH*	upstream
cg00068810	4	75857963	*PARM1*	upstream
cg18197594	10	94334836	*IDE*	upstream
cg21402921	15	27112305	*GABRA5*	UTR5
cg16928454	4	55523124	*KIT*	upstream
cg08939787	2	238767226	*RAMP1*	upstream
cg19408180	21	43809237	*TMPRSS3*	UTR5
cg22439241	21	30366036	*RNF160*	upstream
cg12896271	4	119512856	*LOC729218*	upstream
cg03486287	9	99145998	*SLC35D2*	upstream
cg19909036	15	90294776	*MESP1*	upstream
cg18950481	1	246952889	*LOC149134*	upstream
cg23289456	20	749620	*C20orf54*	upstream
cg16706260	12	27849370	*REP15*	upstream;downstream
cg18444875	8	107460168	*OXR1*	UTR5
cg04602992	17	62009628	*CD79B*	UTR5
cg14558114	2	88469736	*THNSL2*	upstream
cg05224280	17	65372550	*PITPNC1*	upstream
cg11916930	14	50320808	*NEMF*	upstream
cg14993491	1	55504971	*PCSK9*	upstream
cg19154323	2	208890802	*PLEKHM3*	upstream

cg18715057	2	88469819	*THNSL2*	UTR5
cg00523161	2	11888753	*LPIN1*	UTR5
cg05008496	12	26348011	*SSPN*	upstream
cg00919013	8	12613847	*LONRF1*	upstream
cg12819826	19	10216676	*PPAN*	upstream
cg18648167	19	51320867	*MGC45922*	upstream
cg11556592	17	79283300	*C17orf55*	upstream
cg22930588	3	62860710	*CADPS*	UTR5
cg25325723	20	6104886	*FERMT1*	upstream
cg16391973	5	158689566	*UBLCP1*	upstream
cg00592995	20	56196368	*ZBP1*	upstream
cg18909295	5	175223293	*CPLX2*	upstream
cg11245806	2	20252225	*LAPTM4A*	upstream
cg09655253	6	39895417	*MOCS1*	UTR5
cg22181201	11	76571587	*ACER3*	upstream
cg16049600	5	140579298	*PCDHB11*	upstream
cg04316580	6	53530944	*KLHL31*	upstream
cg17695841	11	45825485	*SLC35C1*	upstream
cg09734798	4	68423941	*STAP1*	upstream
cg06436854	16	89043527	*CBFA2T3*	upstream
cg24586758	5	140480200	*PCDHB3*	UTR5
cg10527010	3	10857795	*SLC6A11*	upstream
cg21235373	5	37371915	*NUP155*	upstream
cg01906015	8	63161579	*NKAIN3*	UTR5
cg23128634	5	170288788	*RANBP17*	upstream
cg25744767	7	79764178	*GNAI1*	UTR5
cg15306126	12	46663900	*SLC38A1*	upstream
cg27146131	6	88182107	*SLC35A1*	upstream
cg16589911	1	161184489	*FCER1G*	upstream;downstream
cg01819759	13	79234251	*RNF219*	upstream
cg17496659	1	3568245	*TP73*	upstream
cg09648933	17	62009835	*CD79B*	upstream
cg01376079	11	67070233	*SSH3*	upstream;downstream
cg20626896	12	51442762	*LETMD1*	UTR5
cg04830357	5	140718339	*PCDHGA1*	UTR5
cg15653044	17	4545718	*ALOX15*	upstream
cg14165978	1	40156757	*HPCAL4*	UTR5
cg11037477	4	99851008	*EIF4E*	UTR5
cg07357987	2	223289306	*SGPP2*	UTR5
cg02704217	17	12568507	*MYOCD*	upstream
cg18070959	5	1856726	*LOC101929034*	upstream
cg20648632	6	88182161	*SLC35A1*	upstream

cg10562340	3	46250357	*CCR1*	upstream
cg26980244	8	24772513	*NEFM*	UTR5
cg17945440	8	109096299	*RSPO2*	upstream
cg14189571	4	188916943	*ZFP42*	UTR5
cg13052223	5	140165651	*PCDHA1*	upstream
cg02884181	5	158689508	*UBLCP1*	upstream
cg25742326	1	901449	*PLEKHN1*	upstream;downstream
cg25342674	1	31881983	*SERINC2*	upstream
cg15004182	20	29896584	*DEFB116*	upstream
cg04053572	1	163038665	*RGS4*	UTR5
cg10258721	7	94023442	*COL1A2*	upstream
cg18423935	3	120626667	*STXBP5L*	upstream
cg00193200	3	120626653	*STXBP5L*	upstream
cg08538752	21	10990890	*TPTE*	UTR5
cg07737781	7	73038866	*MLXIPL*	UTR5
cg03469082	4	46126448	*GABRG1*	upstream
cg09703048	14	20584899	*OR4K17*	upstream
cg22150661	8	104032860	*ATP6V1C1*	upstream
cg10494684	17	26645200	*TMEM97*	upstream
cg07590929	16	68270679	*ESRP2*	upstream
cg15858239	8	63161609	*NKAIN3*	UTR5
cg05498618	9	36036451	*RECK*	upstream
cg20165416	10	89264080	*MINPP1*	upstream;downstream
cg05605052	19	23945659	*RPSAP58*	upstream
cg00295572	1	3568236	*TP73*	upstream
cg03774803	2	198650880	*BOLL*	UTR5
cg17393016	17	79283390	*C17orf55*	upstream
cg26642082	1	234508347	*COA6*	upstream;downstream
cg01734112	2	26624760	*C2orf39*	upstream
cg20024110	6	32977708	*HLA-DOA*	upstream
cg25784220	19	58609602	*ZSCAN18*	UTR5
cg10775141	16	30457534	*SEPHS2*	upstream
cg27458987	12	33050050	*PKP2*	upstream
cg23893806	6	143999122	*PHACTR2*	UTR5
cg16312655	12	46663902	*SLC38A1*	upstream
cg22058122	18	6414958	*L3MBTL4*	upstream
cg03878133	2	164593411	*FIGN*	upstream
cg19700376	2	74875760	*M1AP*	upstream
cg11545232	3	156544835	*LEKR1*	UTR5
cg19078576	5	17217877	*BASP1*	UTR5
cg15613567	10	134901497	*GPR123*	upstream
cg26643967	19	36485282	*SDHAF1*	upstream

cg00043095	2	223289277	*SGPP2*	UTR5
cg04621103	6	37321149	*RNF8*	upstream
cg24964103	6	133035379	*VNN1*	upstream
cg27063372	16	616857	*NHLRC4*	upstream
cg01210325	12	2944760	*NRIP2*	upstream
cg21324555	10	72648679	*PCBD1*	upstream
cg24121168	3	58652503	*FAM3D*	UTR5
cg18863595	3	6902845	*GRM7*	UTR5
cg16096646	4	119771931	*SYNPO2*	UTR5
cg27473053	19	50472464	*SIGLEC16*	upstream
cg02964602	2	203130213	*NOP58*	upstream
cg05027516	14	22039204	*OR10G3*	upstream
cg15633390	4	99851211	*EIF4E*	UTR5
cg02105093	17	27070369	*TRAF4*	upstream;downstream
cg17873750	1	116382984	*NHLH2*	UTR5
cg03868206	3	160284293	*KPNA4*	upstream
cg25268605	1	47698518	*TAL1*	upstream
cg19389372	19	36485356	*SDHAF1*	upstream
cg16345226	19	16771394	*TMEM38A*	upstream
cg14972143	4	99851003	*EIF4E*	UTR5
cg09181644	4	141490428	*UCP1*	upstream
cg19192120	11	67070517	*SSH3*	upstream;downstream
cg06096336	2	231989800	*PSMD1*	UTR5
cg18373318	5	53813164	*SNX18*	upstream
cg11461808	7	1126579	*GPER*	UTR5
cg19321979	1	156610966	*BCAN*	upstream
cg16406186	8	110988444	*KCNV1*	upstream
cg26186727	18	70534534	*NETO1*	UTR5
cg21554861	4	99851060	*EIF4E*	UTR5
cg21680327	1	36108225	*PSMB2*	upstream
cg08931687	5	140561245	*PCDHB16*	upstream
cg15132169	6	149805995	*ZC3H12D*	UTR5
cg13303179	3	46759335	*PRSS50*	UTR5
cg11668923	10	128076936	*ADAM12*	UTR5
cg05790550	9	96108842	*C9orf129*	upstream
cg09958192	16	67926607	*PSKH1*	upstream
cg14429906	11	11862429	*USP47*	upstream
cg10555383	8	143859990	*LYNX1*	upstream
cg00210210	6	99798738	*C6orf168*	upstream
cg22721334	19	58609618	*ZSCAN18*	UTR5
cg09678939	6	149806081	*ZC3H12D*	UTR5
cg02165355	5	178368071	*ZNF454*	upstream

cg10368935	10	18240316	*SLC39A12*	upstream
cg15389881	13	113950913	*LAMP1*	upstream
cg22039638	2	70996014	*ADD2*	upstream
cg09416149	6	74364495	*SLC17A5*	upstream
cg17357286	1	210111531	*SYT14*	UTR5
cg24843474	1	241520598	*RGS7*	upstream
cg09153267	15	74500175	*STRA6*	UTR5
cg08383695	6	10886346	*SYCP2L*	upstream
cg26289142	2	74875794	*M1AP*	upstream
cg22418909	8	41166738	*SFRP1*	UTR5
cg13149686	14	20691680	*OR11H6*	upstream
cg15124400	9	124989839	*LHX6*	UTR5
cg15521034	11	124823390	*CCDC15*	upstream
cg22172038	1	231176991	*FAM89A*	upstream
cg07365439	13	19581746	*LINC00442*	upstream
cg17976873	14	23352295	*REM2*	upstream
cg15417244	12	41831558	*PDZRN4*	UTR5
cg12979807	2	55497079	*MTIF2*	upstream
cg20705781	11	67070238	*SSH3*	upstream;downstream
cg02634341	1	179711583	*FAM163A*	UTR5
cg00012522	15	98503952	*ARRDC4*	UTR5
cg01874867	7	94954059	*PON1*	upstream
cg26204079	2	234262348	*DGKD*	upstream
cg15681537	21	22370733	*NCAM2*	UTR5
cg15825059	11	110167871	*RDX*	upstream
cg14480262	14	73703844	*PAPLN*	upstream
cg19384289	2	176994365	*HOXD8*	upstream
cg24946597	8	31497464	*NRG1*	UTR5
cg22879458	5	140566496	*PCDHB9*	upstream;downstream
cg01933073	4	186732942	*SORBS2*	UTR5
cg08855288	8	63161589	*NKAIN3*	UTR5
cg25084878	5	127873711	*FBN2*	UTR5
cg20418550	18	61420051	*SERPINB7*	upstream
cg15842366	6	74364533	*SLC17A5*	upstream
cg13464915	18	25757202	*CDH2*	UTR5
cg19840088	2	149894678	*LYPD6B*	upstream
cg12245770	19	23945761	*RPSAP58*	UTR5
cg00668337	11	64491331	*NRXN2*	upstream
cg06970744	1	110210439	*GSTM2*	upstream
cg05924652	6	39016363	*GLP1R*	upstream
cg10669058	19	19648555	*CILP2*	upstream;downstream
cg22777062	21	38353114	*HLCS*	UTR5

cg03605201	8	91095553	*CALB1*	upstream
cg09557034	7	11872059	*THSD7A*	upstream
cg07482372	11	2951201	*PHLDA2*	upstream
cg03823904	7	80548456	*SEMA3C*	UTR5
cg01349933	14	72398252	*RGS6*	upstream
cg12256845	6	48037248	*PTCHD4*	upstream
cg27403635	5	113698291	*KCNN2*	UTR5
cg26866348	5	191547	*LRRC14B*	upstream
cg06128198	10	128593922	*DOCK1*	upstream
cg12409409	7	130125963	*MEST*	upstream;downstream
cg22190023	5	140552347	*PCDHB7*	UTR5
cg09150559	5	140571517	*PCDHB10*	upstream;downstream
cg26422488	5	16935950	*MYO10*	UTR5
cg01243879	6	166722248	*PRR18*	upstream
cg17725398	19	58962387	*ZNF324B*	upstream
cg10281770	17	80606947	*WDR45L*	upstream
cg26785346	4	186732936	*SORBS2*	UTR5
cg04100843	4	4860655	*MSX1*	upstream
cg25546811	7	93550752	*GNG11*	upstream
cg03015952	19	55987366	*ZNF628*	upstream
cg16015423	19	15217974	*SYDE1*	upstream
cg26141335	7	130125976	*MEST*	upstream;downstream
cg03809622	14	104552016	*ASPG*	upstream
cg11647681	5	140810161	*PCDHGA4*	UTR5
cg09102079	1	53164386	*COA7*	upstream
cg23180489	8	143859786	*LYNX1*	upstream
cg04892170	10	128076910	*ADAM12*	UTR5
cg26044490	12	16761419	*LMO3*	upstream
cg15138396	2	26624450	*C2orf39*	upstream
cg16848272	1	1510991	*SSU72*	upstream
cg04256347	4	141677674	*TBC1D9*	upstream
cg25273602	1	29062859	*YTHDF2*	upstream
cg01747364	11	66233628	*PELI3*	upstream
cg25555787	6	41196139	*TREML4*	UTR5
cg14007128	17	72864722	*FDXR*	UTR5
cg15482931	15	27019448	*GABRB3*	upstream
cg03906910	5	142814388	*NR3C1*	UTR5
cg20881888	3	50383079	*ZMYND10*	UTR5
cg17108629	11	87908805	*RAB38*	upstream
cg26051413	11	2292902	*ASCL2*	upstream
cg22620414	3	10806133	*LOC285370*	upstream
cg01557027	11	24518448	*LUZP2*	upstream

cg25092283	10	97321585	*SORBS1*	upstream
cg19284039	11	2292895	*ASCL2*	upstream
cg09319843	18	25757569	*CDH2*	upstream
cg13735282	4	11430724	*HS3ST1*	upstream
cg16818628	10	50604006	*DRGX*	UTR5
cg26317209	12	133187136	*LRCOL1*	upstream
cg09843519	5	125931316	*ALDH7A1*	upstream
cg00606190	12	16761702	*LMO3*	upstream
cg03321508	20	25039707	*ACSS1*	upstream
cg20343567	1	249121271	*SH3BP5L*	upstream;downstream
cg21147238	22	44838755	*LOC101927526*	upstream
cg26811372	5	140772182	*PCDHGA4*	UTR5
cg26886277	2	86566099	*REEP1*	upstream
cg24715245	4	41258794	*UCHL1*	upstream
cg18417245	5	140579296	*PCDHB11*	upstream
cg11481675	8	32504965	*NRG1*	UTR5
cg19225512	10	72648693	*PCBD1*	upstream
cg17741128	6	109704936	*CD164*	upstream
cg12943364	16	30418256	*ZNF771*	upstream
cg20670160	2	189156621	*GULP1*	UTR5
cg18342119	4	186733011	*SORBS2*	UTR5
cg04565008	11	67418140	*ACY3*	upstream
cg09905935	18	20713999	*CABLES1*	upstream
cg14779329	11	130786720	*SNX19*	upstream
cg06241208	11	30344200	*C11orf46*	upstream
cg00160911	14	70041888	*CCDC177*	upstream
cg24977260	1	97186694	*PTBP2*	upstream
cg04471507	1	26233376	*STMN1*	upstream
cg17794788	2	238767409	*RAMP1*	upstream
cg26866168	5	140528785	*PCDHB6*	upstream
cg15111469	5	121415023	*LOX*	upstream
cg17330251	7	94953956	*PON1*	UTR5
cg24378421	8	11141365	*MTMR9*	upstream
cg00615271	19	14799963	*ZNF333*	upstream
cg03387723	1	41708464	*SCMH1*	upstream
cg01926269	2	25391670	*POMC*	UTR5
cg26622291	5	140710237	*PCDHGA1*	upstream
cg00399059	8	143859774	*LYNX1*	upstream
cg19922137	1	210111561	*SYT14*	UTR5
cg06912990	4	159131518	*TMEM144*	UTR5
cg10593400	1	86044176	*DDAH1*	upstream
cg09937190	20	15177509	*MACROD2*	UTR5

cg15351009	5	191486	*LRRC14B*	upstream
cg09866366	3	183903315	*ABCF3*	upstream
cg18354264	1	226411008	*MIXL1*	upstream
cg03496709	10	29698460	*LOC387647*	upstream
cg26248284	4	41362853	*LIMCH1*	UTR5
cg09368486	2	208890779	*PLEKHM3*	upstream

MAPINFO means genomic location; UCSC_Ref Gene_Group_Anovar represents gene region where the probe is located based on anovar annotation. *CHR* Chromosome; *DMPs* DNA methylation positions; *5'UTR* 5'untranslated region
