# Supplementary material for: Association of DNA Methylation Patterns in 7 Novel Genes With Ischemic Stroke in the Northern Chinese Population
Source: Front Genet. 2022 Apr 11;13:844141. doi: 10.3389/fgene.2022.844141 (PMC9035884; doi:10.3389/fgene.2022.844141)
Supplement: Supplementary file 3 [file DataSheet4.PDF]

Additional file 4

203 differentially methylated CpG sites

site	Group difference	*P* value	Adjust *P* value
CDH2_1_36	-0.0144	*P*<0.0001*	*P*<0.0001*
CDH2_1_40	-0.0100	*P*<0.0001*	*P*<0.0001*
CDH2_1_45	-0.0146	*P*<0.0001*	*P*<0.0001*
CDH2_1_50	-0.0123	*P*<0.0001*	*P*<0.0001*
CDH2_1_59	-0.0098	*P*<0.0001*	*P*<0.0001*
CDH2_1_61	-0.0088	*P*<0.0001*	*P*<0.0001*
CDH2_1_64	-0.0130	*P*<0.0001*	*P*<0.0001*
CDH2_1_73	-0.0165	*P*<0.0001*	*P*<0.0001*
CDH2_1_76	-0.0102	*P*<0.0001*	*P*<0.0001*
CDH2_1_82	-0.0255	*P*<0.0001*	*P*<0.0001*
CDH2_1_85	-0.0147	*P*<0.0001*	*P*<0.0001*
CDH2_1_87	-0.0075	*P*<0.0001*	*P*=0.0002*
CDH2_1_90	-0.0203	*P*<0.0001*	*P*<0.0001*
CDH2_1_94	-0.0161	*P*<0.0001*	*P*<0.0001*
CDH2_1_105	-0.0110	*P*<0.0001*	*P*<0.0001*
CDH2_1_113	-0.0117	*P*<0.0001*	*P*<0.0001*
CDH2_1_130	-0.0036	*P*<0.0001*	*P*=0.0002*
CDH2_1_136	-0.0097	*P*<0.0001*	*P*<0.0001*
CDH2_1_144	-0.0048	*P*<0.0001*	*P*<0.0001*
CDH2_1_146	-0.0027	*P*<0.0001*	*P*=0.0002*
CDH2_1_151	-0.0062	*P*<0.0001*	*P*<0.0001*
CDH2_2_22	-0.0112	*P*<0.0001*	*P*<0.0001*
CDH2_2_25	-0.0091	*P*<0.0001*	*P*<0.0001*
CDH2_2_44	-0.0162	*P*<0.0001*	*P*<0.0001*
CDH2_2_47	-0.0052	*P*<0.0001*	*P*<0.0001*
CDH2_2_55	-0.0093	*P*<0.0001*	*P*<0.0001*
CDH2_2_57	-0.0102	*P*<0.0001*	*P*<0.0001*
CDH2_2_74	-0.0139	*P*<0.0001*	*P*<0.0001*
CDH2_2_79	-0.0132	*P*<0.0001*	*P*<0.0001*
CDH2_2_84	-0.0204	*P*<0.0001*	*P*<0.0001*
CDH2_2_87	-0.0109	*P*<0.0001*	*P*<0.0001*
CDH2_2_92	-0.0156	*P*<0.0001*	*P*<0.0001*
CDH2_2_98	-0.0112	*P*<0.0001*	*P*<0.0001*
CDH2_2_102	-0.0113	*P*<0.0001*	*P*<0.0001*
CDH2_2_109	-0.0098	*P*<0.0001*	*P*<0.0001*
CDH2_2_111	-0.0084	*P*<0.0001*	*P*<0.0001*
CDH2_2_117	-0.0103	*P*<0.0001*	*P*<0.0001*
CDH2_2_121	-0.0107	*P*<0.0001*	*P*<0.0001*
CDH2_2_131	-0.0147	*P*<0.0001*	*P*<0.0001*

CDH2_3_25	-0.0168	*P*=0.0001*	*P*=0.0014*
CDH2_3_27	-0.0127	*P*=0.0065*	*P*=0.0443*
CDH2_3_30	-0.0163	*P*=0.0014*	*P*=0.0028*
CDH2_3_37	-0.0143	*P*=0.0025*	*P*=0.0015*
CDH2_3_39	-0.0162	*P*=0.0012*	*P*=0.0080*
CDH2_3_55	-0.0094	*P*=0.0080*	*P*=0.0248*
CDH2_3_65	-0.0076	*P*=0.0316*	*P*=0.0064*
CDH2_3_69	-0.0112	*P*=0.0026*	*P*=0.0581
CDH2_3_75	-0.0099	*P*=0.0100*	*P*=0.1129
CDH2_3_92	-0.0094	*P*=0.0237*	*P*=0.0420*
CDH2_3_94	-0.0140	*P*=0.0001*	*P*=0.0017*
CDH2_3_97	-0.0215	*P*<0.0001*	*P*=0.0011*
CDH2_3_102	-0.0142	*P*=0.0027*	*P*=0.0074*
CDH2_3_108	-0.0135	*P*=0.0018*	*P*=0.0325*
CDH2_3_115	-0.0180	*P*=0.0039*	*P*=0.0168*
CDH2_3_121	-0.0108	*P*=0.0116*	*P*=0.0355*
CDH2_3_138	-0.0329	*P*<0.0001*	*P*=0.0006*
CDH2_3_141	-0.0198	*P*=0.0006*	*P*=0.0199*
CDH2_3_146	-0.0310	*P*<0.0001*	*P*=0.0003*
CDH2_3_148	-0.0250	*P*<0.0001*	*P*=0.0009*
CDH2_3_151	-0.0262	*P*<0.0001*	*P*=0.0044*
CDH2_3_153	-0.0160	*P*=0.0038*	*P*=0.0101*
CDH2_3_156	-0.0312	*P*<0.0001*	*P*=0.0005*
CDH2_3_158	-0.0213	*P*=0.0002*	*P*=0.0022*
CDH2_3_160	-0.0196	*P*=0.0014*	*P*=0.0086*
CDH2_3_164	-0.0157	*P*=0.0105*	*P*=0.0298*
CDH2_3_173	-0.0138	*P*=0.0122*	*P*=0.3451
CDH2_3_189	-0.0139	*P*=0.0060*	*P*=0.0179*
CDH2_3_193	-0.0156	*P*=0.0093*	*P*=0.0737
CDH2_3_200	-0.0133	*P*=0.0086*	*P*=0.0381*
CDH2_3_214	-0.0079	*P*=0.0493*	*P*=0.0112*
CDH2_3_232	-0.0072	*P*=0.0463*	*P*=0.2456
CDH2_4_29	-0.0074	*P*<0.0001*	*P*=0.0010*
CDH2_4_34	-0.0060	*P*=0.0007*	*P*=0.0026*
CDH2_4_40	-0.0084	*P*<0.0001*	*P*=0.0014*
CDH2_4_60	-0.0108	*P*<0.0001*	*P*=0.0001*
CDH2_4_91	-0.0049	*P*=0.0002*	*P*=0.0008*
CDH2_4_128	-0.0107	*P*<0.0001*	*P*<0.0001*
CDH2_4_130	-0.0082	*P*<0.0001*	*P*<0.0001*
CDH2_4_154	-0.0152	*P*<0.0001*	*P*=0.0002*
CDH2_4_158	-0.0145	*P*<0.0001*	*P*=0.0001*
CDH2_4_170	-0.0134	*P*<0.0001*	*P*<0.0001*
CDH2_4_180	-0.0134	*P*<0.0001*	*P*<0.0001*
CDH2_4_185	-0.0114	*P*<0.0001*	*P*<0.0001*

PCDHB10_5_34	-0.0108	*P*=0.0260*	*P*=0.0493*
PCDHB10_5_64	-0.0166	*P*=0.0011*	*P*=0.0039*
PCDHB10_5_71	-0.0244	*P*<0.0001*	*P*=0.0002*
PCDHB10_5_78	-0.0246	*P*<0.0001*	*P*<0.0001*
PCDHB10_5_86	-0.0283	*P*<0.0001*	*P*<0.0001*
PCDHB10_5_92	-0.0265	*P*<0.0001*	*P*<0.0001*
PCDHB10_5_114	-0.0274	*P*<0.0001*	*P*=0.0003*
PCDHB10_5_119	-0.0346	*P*<0.0001*	*P*<0.0001*
PCDHB10_5_124	-0.0353	*P*<0.0001*	*P*<0.0001*
PCDHB10_5_137	-0.0235	*P*<0.0001*	*P*=0.0009*
PCDHB10_5_152	-0.02137	*P*=0.0002*	*P*=0.0021*
PCDHB10_5_161	-0.0256	*P*<0.0001*	*P*<0.0001*
PCDHB10_5_170	-0.0152	*P*=0.0007*	*P*=0.0020*
PCDHB10_5_187	-0.0246	*P*<0.0001*	*P*=0.0016*
PCDHB10_5_205	-0.0253	*P*<0.0001*	*P*=0.0007*
PCDHB10_5_221	-0.0260	*P*<0.0001*	*P*=0.0007*
PCDHB10_5_227	-0.0354	*P*<0.0001*	*P*<0.0001*
PCDHB10_5_233	-0.0300	*P*<0.0001*	*P*<0.0001*
PCDHB11_6_45	-0.0208	*P*=0.0008*	*P*=0.0023*
PCDHB11_6_51	-0.0169	*P*=0.0264*	*P*=0.0117*
PCDHB11_6_73	-0.0238	*P*=0.0008*	*P*=0.0051*
PCDHB11_6_78	-0.0312	*P*<0.0001*	*P*=0.0002*
PCDHB11_6_83	-0.0336	*P*<0.0001*	*P*=0.0003*
PCDHB11_6_96	-0.0247	*P*=0.0003*	*P*=0.0055*
PCDHB11_6_111	-0.0237	*P*=0.0001*	*P*=0.0005*
PCDHB11_6_120	-0.0283	*P*<0.0001*	*P*=0.0004*
PCDHB11_6_129	-0.0252	*P*<0.0001*	*P*=0.0003*
PCDHB11_6_146	-0.0183	*P*=0.0021*	*P*=0.0462*
PCDHB11_6_153	-0.0246	*P*<0.0001*	*P*=0.0006*
PCDHB11_6_158	-0.0218	*P*<0.0001*	*P*=0.0017*
PCDHB11_6_174	-0.0134	*P*=0.0044*	*P*=0.0412*
PCDHB11_6_180	-0.0287	*P*<0.0001*	*P*=0.0001*
PCDHB11_6_192	-0.0247	*P*=0.0030*	*P*=0.0009*
PCDHB14_7_27	-0.0260	*P*<0.0001*	*P*=0.0052*
PCDHB14_7_33	-0.0116	*P*=0.0021*	*P*=0.0967
PCDHB14_7_66	-0.0174	*P*=0.0004*	*P*=0.0034*
PCDHB14_7_68	-0.0132	*P*=0.0102*	*P*=0.0204*
PCDHB14_7_72	-0.0222	*P*=0.0002*	*P*=0.0024*
PCDHB14_8_81	-0.0252	*P*=0.0360*	*P*=0.2734
PCDHB14_8_87	-0.0253	*P*=0.0293*	*P*=0.8661
PCDHB14_8_109	-0.0497	*P*=0.0005*	*P*=0.0973
PCDHB14_8_114	-0.0230	*P*=0.0437*	*P*=0.2078
PCDHB14_8_119	-0.0396	*P*=0.0014*	*P*=0.0015*
PCDHB14_8_132	-0.0293	*P*=0.0215*	*P*=0.0186*

PCDHB14_8_147	-0.0513	*P*=0.0002*	*P*=0.0159*
PCDHB14_8_150	-0.0201	*P*<0.0001*	*P*=0.0005*
PCDHB14_8_182	-0.0306	*P*=0.0194*	*P*=0.2059
PCDHB14_8_194	-0.0397	*P*=0.0010*	*P*=0.5804
PCDHB14_8_216	-0.0350	*P*=0.0067*	*P*=0.2225
PCDHB16_10_23	-0.0225	*P*=0.0263*	*P*=0.1344
PCDHB16_10_38	-0.0316	*P*<0.0001*	*P*=0.0005*
PCDHB16_10_45	-0.0320	*P*=0.0026*	*P*=0.0101*
PCDHB16_10_50	-0.0554	*P*<0.0001*	*P*=0.0014*
PCDHB16_10_57	-0.0304	*P*=0.0040*	*P*=0.0048*
PCDHB16_10_61	-0.036	*P*=0.0006*	*P*=0.0008*
PCDHB16_10_63	-0.0339	*P*=0.0002*	*P*=0.0079*
PCDHB16_10_113	-0.0269	*P*=0.0099*	*P*=0.0133*
PCDHB16_10_116	-0.0333	*P*=0.0022*	*P*=0.0212*
PCDHB16_10_118	-0.0322	*P*=0.0036*	*P*=0.0277*
PCDHB16_10_122	-0.0326	*P*=0.0032*	*P*=0.0285*
PCDHB16_10_137	-0.0339	*P*=0.0005*	*P*=0.0033*
PCDHB16_10_149	-0.0265	*P*=0.0123*	*P*=0.0411*
PCDHB16_10_170	-0.0257	*P*=0.0041*	*P*=0.0019*
PCDHB3_11_53	-0.0283	*P*=0.0059*	*P*=0.0023*
PCDHB3_11_55	-0.0200	*P*=0.0359*	*P*=0.0287*
PCDHB3_11_62	-0.0243	*P*=0.0145*	*P*=0.0158*
PCDHB3_11_94	-0.0353	*P*=0.0005*	*P*=0.0320*
PCDHB3_11_133	-0.0218	*P*=0.0189*	*P*=0.0385*
PCDHB3_11_141	-0.0278	*P*=0.0047*	*P*=0.0038*
PCDHB3_11_157	-0.0243	*P*=0.0146*	*P*=0.0226*
PCDHB3_12_24	-0.0295	*P*<0.0001*	*P*<0.0001*
PCDHB3_12_46	-0.0328	*P*<0.0001*	*P*<0.0001*
PCDHB3_12_51	-0.0358	*P*<0.0001*	*P*<0.0001*
PCDHB3_12_58	-0.0250	*P*<0.0001*	*P*<0.0001*
PCDHB3_12_62	-0.0294	*P*<0.0001*	*P*<0.0001*
PCDHB3_12_64	-0.0338	*P*<0.0001*	*P*<0.0001*
PCDHB3_12_72	-0.0174	*P*=0.0040*	*P*=0.0029*
PCDHB3_12_112	-0.0137	*P*=0.0008*	*P*=0.0186*
PCDHB3_12_114	-0.0332	*P*<0.0001*	*P*<0.0001*
PCDHB3_12_117	-0.0296	*P*<0.0001*	*P*<0.0001*
PCDHB3_12_119	-0.0296	*P*<0.0001*	*P*=0.0002*
PCDHB3_12_123	-0.0340	*P*<0.0001*	*P*<0.0001*
PCDHB3_12_138	-0.0367	*P*<0.0001*	*P*<0.0001*
PCDHB3_12_143	-0.0213	*P*=0.0006*	*P*=0.0092*
PCDHB3_12_150	-0.0236	*P*=0.0001*	*P*=0.0005*
PCDHB3_12_160	-0.0238	*P*=0.0004*	*P*=0.0012*
PCDHB3_12_168	-0.0171	*P*=0.0003*	*P*=0.0009*
PCDHB3_12_171	-0.0232	*P*<0.0001*	*P*<0.0001*

PCDHB3_12_180	-0.0190	*P*=0.0018*	*P*=0.0083*
PCDHB3_13_177	-0.0163	*P*=0.0479*	*P*=0.9137
PCDHB6_14_107	-0.0264	*P*=0.0252*	*P*=0.0406*
PCDHB6_15_101	-0.0243	*P*=0.0334*	*P*=0.1055
PCDHB9_16_25	-0.0281	*P*<0.0001*	*P*=0.0001*
PCDHB9_16_40	-0.0250	*P*<0.0001*	*P*<0.0001*
PCDHB9_16_47	-0.0281	*P*<0.0001*	*P*=0.0001*
PCDHB9_16_52	-0.0354	*P*<0.0001*	*P*<0.0001*
PCDHB9_16_59	-0.0239	*P*<0.0001*	*P*=0.0009*
PCDHB9_16_63	-0.0266	*P*<0.0001*	*P*<0.0001*
PCDHB9_16_73	-0.0156	*P*=0.0082*	*P*=0.0048*
PCDHB9_16_115	-0.0354	*P*<0.0001*	*P*<0.0001*
PCDHB9_16_118	-0.0356	*P*<0.0001*	*P*<0.0001*
PCDHB9_16_120	-0.0329	*P*<0.0001*	*P*<0.0001*
PCDHB9_16_124	-0.0354	*P*<0.0001*	*P*<0.0001*
PCDHB9_16_139	-0.0385	*P*<0.0001*	*P*<0.0001*
PCDHB9_16_144	-0.0228	*P*=0.0003*	*P*=0.0033*
PCDHB9_16_161	-0.0254	*P*=0.0001*	*P*=0.0004*
PCDHB9_16_172	-0.0208	*P*<0.0001*	*P*=0.0006*
PCDHB9_16_181	-0.0197	*P*=0.0008*	*P*=0.0105*
PCDHB9_17_65	-0.0209	*P*=0.0232*	*P*=0.0553
PCDHB9_17_80	-0.0294	*P*=0.0012*	*P*=0.0169*
PCDHB9_17_108	-0.0326	*P*=0.0013*	*P*=0.0014*
PCDHB9_17_113	-0.0304	*P*=0.0022*	*P*=0.0177*
PCDHB9_17_118	-0.0381	*P*=0.0004*	*P*=0.0023*
PCDHB9_17_146	-0.0392	*P*<0.0001*	*P*=0.0071*
PCDHB9_17_155	-0.0333	*P*=0.0007*	*P*=0.0011*
PCDHB9_17_181	-0.0320	*P*=0.0008*	*P*=0.0175*
PCDHB9_17_193	-0.0283	*P*=0.0044*	*P*=0.1241
PCDHB9_17_209	-0.0208	*P*=0.0323*	*P*=0.2512
PCDHB9_17_215	-0.0551	*P*<0.0001*	*P*<0.0001*
PCDHB9_17_221	-0.0372	*P*=0.0013*	*P*=0.0114*

Adjusted factors: smoking, drinking, previous medical history of hypertension and diabetes mellitus, and plasma lipid levels (total triglyceride, total cholesterol, high-density lipoprotein, and low-density lipoprotein). *Statistically significant difference (*P*<0.05). *CpG* cytosine phosphate guanine
