# Supplementary material for: Association of DNA Methylation Patterns in 7 Novel Genes With Ischemic Stroke in the Northern Chinese Population
Source: Front Genet. 2022 Apr 11;13:844141. doi: 10.3389/fgene.2022.844141 (PMC9035884; doi:10.3389/fgene.2022.844141)
Supplement: Supplementary file 4 [file DataSheet6.PDF]

Additional file 6

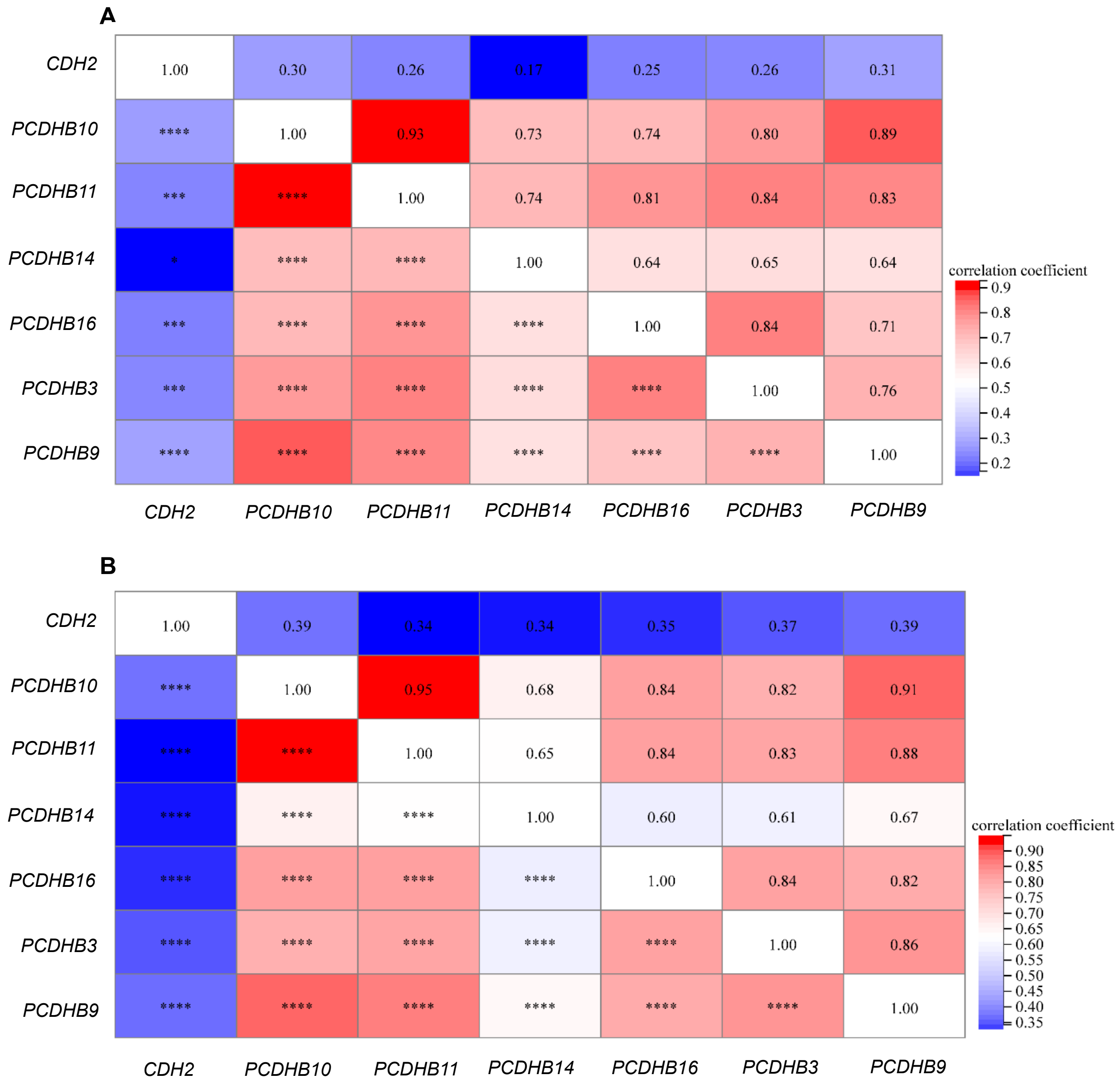

Correlation analysis of the methylation level of the 7 genes in case (A) and control group (B). The upper right corner of the picture is the correlation coefficient, and the lower left corner is the significance of the correlation. *Statistically significant difference (P<0.05). ***Statistically significant difference (P<0.001). ****Statistically significant difference (P<0.0001).
