# Supplementary material for: Association of DNA Methylation Patterns in 7 Novel Genes With Ischemic Stroke in the Northern Chinese Population
Source: Front Genet. 2022 Apr 11;13:844141. doi: 10.3389/fgene.2022.844141 (PMC9035884; doi:10.3389/fgene.2022.844141)
Supplement: Supplementary file 7 [file DataSheet3.PDF]

Additional file 3

10 CpG sites in 850K BeadChip and replication stage.

Target	Gene Name	Group difference	*P* value	Methylation type in IS
850K array				
cg08931687	*PCDHB16*	-0.1711	*P*=0.0307*	Hypomethylated
cg09150559	*PCDHB10*	-0.2068	*P*=0.0390*	Hypomethylated
cg09319843	*CDH2*	-0.1519	*P*=0.0438*	Hypomethylated
cg13464915	*CDH2*	-0.1600	*P*=0.0357*	Hypomethylated
cg18417245	*PCDHB11*	-0.1026	*P*=0.0451*	Hypomethylated
cg16049600	*PCDHB11*	-0.1999	*P*=0.0182*	Hypomethylated
cg21522026	*PCDHB14*	-0.1275	*P*=0.0059*	Hypomethylated
cg22879458	*PCDHB9*	-0.1314	*P*=0.0344*	Hypomethylated
cg24586758	*PCDHB3*	-0.1300	*P*=0.0186*	Hypomethylated
cg26866168	*PCDHB6*	-0.1397	*P*=0.0470*	Hypomethylated
Replication in 20 paired samples				
cg08931687	*PCDHB16*	-0.0970	*P*=0.0280*	Hypomethylated
cg09150559	*PCDHB10*	-0.1121	*P*=0.0042*	Hypomethylated
cg09319843	*CDH2*	-0.0183	*P*=0.1222	Hypomethylated
cg13464915	*CDH2*	-0.0244	*P*=0.0374*	Hypomethylated
cg18417245	*PCDHB11*	-0.0687	*P*=0.0683	Hypomethylated
cg16049600	*PCDHB11*	-0.1226	*P*=0.0175*	Hypomethylated
cg21522026	*PCDHB14*	-0.0257	*P*=0.1509	Hypomethylated
cg22879458	*PCDHB9*	-0.1043	*P*=0.0035*	Hypomethylated
cg24586758	*PCDHB3*	-0.0499	*P*=0.0570	Hypomethylated
cg26866168	*PCDHB6*	-0.0556	*P*=0.0636	Hypomethylated

AS the sample size is small and the samples are marched, we did not correct for confounding factors.

*Statistically significant difference (*P*<0.05). *CpG* cytosine phosphate guanine; *IS* ischemic stroke
