# Supplementary material for: Association of DNA Methylation Patterns in 7 Novel Genes With Ischemic Stroke in the Northern Chinese Population
Source: Front Genet. 2022 Apr 11;13:844141. doi: 10.3389/fgene.2022.844141 (PMC9035884; doi:10.3389/fgene.2022.844141)
Supplement: Supplementary file 8 [file DataSheet1.PDF]

Additional file 1

Information of primers used in MethylTargetTM assay.

Gene	Target	Forward-Seqence	Reverse-Seqence	CHR
CDH2	*CDH2_1*	GAGTGGYGGGATTGTTGTTTT	AATAACRCTCCCCAAAAACTCC	18
CDH2	*CDH2_2*	TTTATTTGTYGGGGYGGTGTT	AACCAATCRAAAACCACCAAAC	18
CDH2	*CDH2_3*	TTGTTGGYGGTTTTGTTTTAGG	CCCTAAAACCCCRCCAAAA	18
CDH2	*CDH2_4*	TTTTGTTYGGTTGTTTGTGTTTT	AATTAAACTACCCCCRAACTAAAAAC	18
PCDHB10	*PCDHB10_5*	GGTAGTTTTGGGATAGGGTTTTAG	TCCCAACCCTACCTACCTCTC	5
PCDHB11	*PCDHB11_6*	AGATGTTTTGGAAAGGGGTTTTT	CTTCTCCCAACCCTACCTACC	5
PCDHB14	*PCDHB14_7*	GTTGGTGTTTGAGTTTTTGTTTGT	TCACAAACTTAAAAACACCCAAAC	5
PCDHB14	*PCDHB14_8*	TTTTGGGATAGGGTTTYGGTGT	AACCCTACCTACCRCTCCCTAAAAC	5
PCDHB16	*PCDHB16_9*	GTTGTTTGTGTTGATGGAGATTAGG	CACCCAAACTAACAACACATCTC	5
PCDHB16	*PCDHB16_10*	GGTAGGAAGGGTTGGGAGA	TACCAACTACTCAAAACCACRAAAC	5
PCDHB3	*PCDHB3_11*	TTTGGATGTTTATTTGGGTTGTTTT	AAACAACCTCCAAAACTACACTATCAC	5
PCDHB3	*PCDHB3_12*	AGGTAGGTAGGGTTGGGAGAAG	TACCAACTACTCAAAACCACRAAAC	5
PCDHB3	*PCDHB3_13*	GTTTTAGTTTTTTAAGTAGTTGGGATTATAGG	AAATATCTTTCTCCTATATCATCATACAAATAAC	5
PCDHB6	*PCDHB6_14*	GGTTGTTTTTAGGGATTTGTGTTTT	ATCAACTCCAACCCTCACTTC	5
PCDHB6	*PCDHB6_15*	ATTAGGGAAGAGAGGGGTAGGT	ACACCAAAACTAAAAACCCAACAA	5
PCDHB9	*PCDHB9_16*	GAGGTAGGTAGGGTTGGGAGAAG	ACCAACTACTCAAAACCACRAAAC	5
PCDHB9	*PCDHB9_17*	TTTGGAATAGGGTTTYGGTGT	ACTTCTCCCAACCCTACCTACCT	5

CHR Chromosome.
