# Supplementary material for: Association of DNA Methylation Patterns in 7 Novel Genes With Ischemic Stroke in the Northern Chinese Population
Source: Front Genet. 2022 Apr 11;13:844141. doi: 10.3389/fgene.2022.844141 (PMC9035884; doi:10.3389/fgene.2022.844141)
Supplement: Supplementary file 9 [file DataSheet5.PDF]

Additional file 5

Validation of 17 CpG islands in candidate genes.

Target	Group difference	*P* value	Adjust *P* value	Adjust FDR *P* value
CDH2_1	-0.0116	*P*<0.0001*	*P*<0.0001*	*P*<0.0001*
CDH2_2	-0.0118	*P*<0.0001*	*P*<0.0001*	*P*<0.0001*
CDH2_3	-0.0148	*P*<0.0001*	*P*=0.0006*	*P*=0.0015*
CDH2_4	-0.0097	*P*<0.0001*	*P*<0.0001*	*P*<0.0001*
PCDHB10_5	-0.0228	*P*<0.0001*	*P*<0.0001*	*P*=0.0001*
PCDHB11_6	-0.0231	*P*<0.0001*	*P*=0.0001*	*P*=0.0005*
PCDHB14_7	-0.0119	*P*=0.0087*	*P*=0.0562	*P*=0.0975
PCDHB14_8	-0.0249	*P*<0.0001*	*P*=0.0163*	*P*=0.0326*
PCDHB16_9	-0.0057	*P*=0.4393	*P*=0.2500	*P*=0.3824
PCDHB16_10	-0.0267	*P*<0.0001*	*P*=0.0004*	*P*=0.0011*
PCDHB3_11	-0.0162	*P*=0.0170*	*P*=0.0187*	*P*=0.0348*
PCDHB3_12	-0.0268	*P*<0.0001*	*P*<0.0001*	*P*<0.0001*
PCDHB3_13	-0.0028	*P*=0.4767	*P*=0.9054	*P*=0.9056
PCDHB6_14	-0.0083	*P*=0.4173	*P*=0.4738	*P*=0.5866
PCDHB6_15	-0.0095	*P*=0.2516	*P*=0.1459	*P*=0.2372
PCDHB9_16	-0.0281	*P*<0.0001*	*P*<0.0001*	*P*=0.0001*
PCDHB9_17	-0.0247	*P*<0.0001*	*P*=0.0010*	*P*=0.0023*

Adjusted factors: smoking, drinking, previous medical history of hypertension and diabetes mellitus, and plasma lipid levels (total triglyceride, total cholesterol, high-density lipoprotein, and low-density lipoprotein). *Statistically significant difference ($P<0.05$). *CpG* cytosine phosphate guanine
