# Supplementary material for: Association of DNA Methylation Patterns in 7 Novel Genes With Ischemic Stroke in the Northern Chinese Population
Source: Front Genet. 2022 Apr 11;13:844141. doi: 10.3389/fgene.2022.844141 (PMC9035884; doi:10.3389/fgene.2022.844141)
Supplement: Supplementary file 10 [file DataSheet8.PDF]

Additional file 8

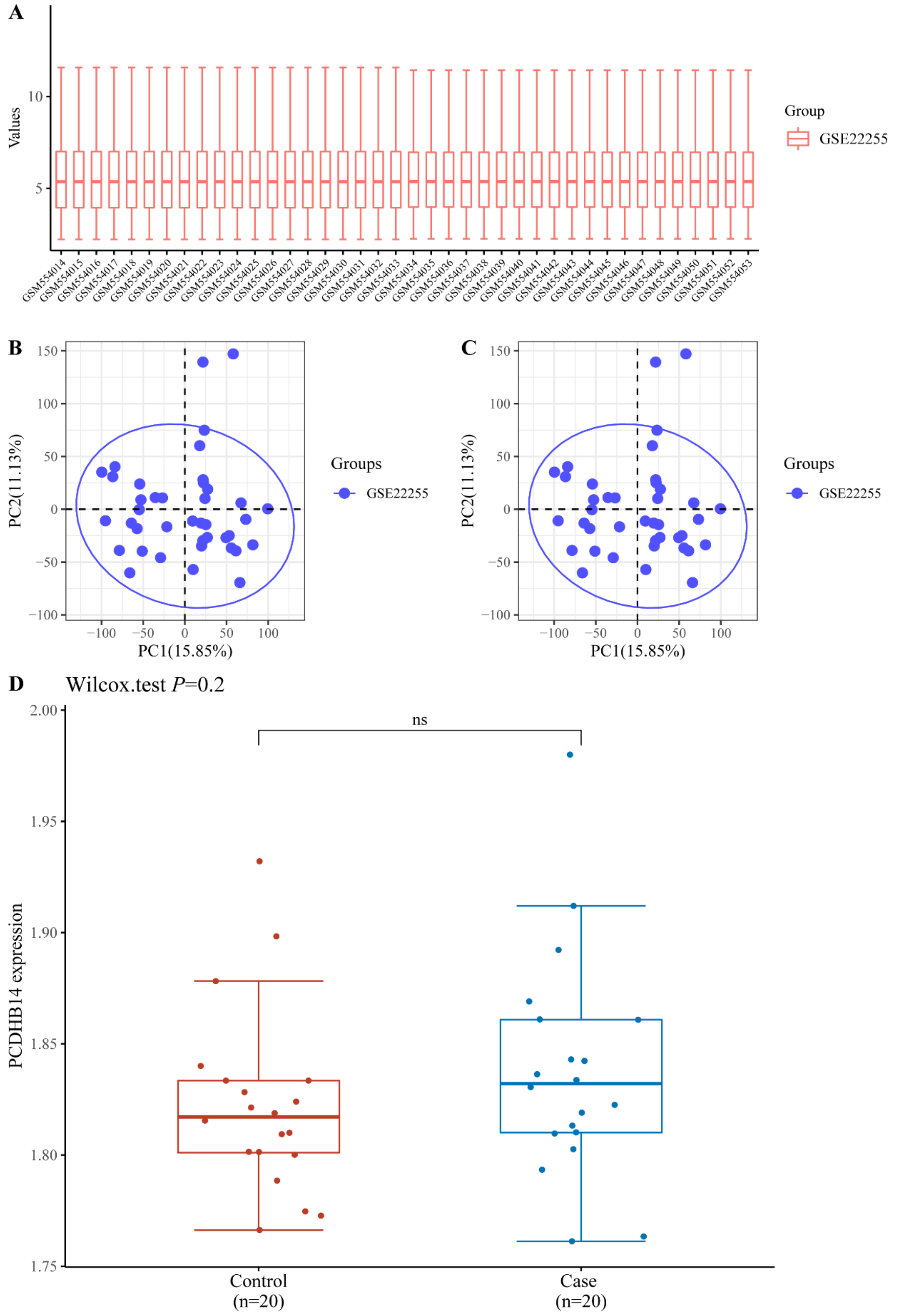

Validation of the mRNA expression of *PCDHB14* gene in GSE22255. A: Box plots after data standardization; B: Principal component analysis without batch removal; C: Principal component analysis after batch removal; D: mRNA expression of the *PCDHB14* gene in the control and case (IS) groups. Not significant abbreviated as ns (*P*>0.05).
